# Anaerobic Sulfur Oxidation Underlies Adaptation of a Chemosynthetic Symbiont to Oxic-Anoxic Interfaces

**DOI:** 10.1128/mSystems.01186-20

**Published:** 2021-05-26

**Authors:** Gabriela F. Paredes, Tobias Viehboeck, Raymond Lee, Marton Palatinszky, Michaela A. Mausz, Siegfried Reipert, Arno Schintlmeister, Andreas Maier, Jean-Marie Volland, Claudia Hirschfeld, Michael Wagner, David Berry, Stephanie Markert, Silvia Bulgheresi, Lena König

**Affiliations:** a University of Vienna, Department of Functional and Evolutionary Ecology, Environmental Cell Biology Group, Vienna, Austria; b University of Vienna, Center for Microbiology and Environmental Systems Science, Division of Microbial Ecology, Vienna, Austria; c Washington State University, School of Biological Sciences, Pullman, Washington, USA; d University of Warwick, School of Life Sciences, Coventry, United Kingdom; e University of Vienna, Core Facility Cell Imaging and Ultrastructure Research, Vienna, Austria; f University of Vienna, Center for Microbiology and Environmental Systems Science, Large-Instrument Facility for Environmental and Isotope Mass Spectrometry, Vienna, Austria; g University of Vienna, Faculty of Geosciences, Geography, and Astronomy, Department of Geography and Regional Research, Geoecology, Vienna, Austria; h University of Greifswald, Institute of Pharmacy, Department of Pharmaceutical Biotechnology, Greifswald, Germany; i Aalborg University, Department of Chemistry and Bioscience, Aalborg, Denmark; j Joint Microbiome Facility of the Medical University of Vienna and the University of Vienna, Vienna, Austria; Vanderbilt University

**Keywords:** *Gammaproteobacteria*, Thiosymbion, anoxia, chemosynthesis, sulfur oxidation, symbiosis, thiotrophic bacteria

## Abstract

Chemosynthetic symbioses occur worldwide in marine habitats, but comprehensive physiological studies of chemoautotrophic bacteria thriving on animals are scarce. Stilbonematinae are coated by thiotrophic *Gammaproteobacteria*. As these nematodes migrate through the redox zone, their ectosymbionts experience varying oxygen concentrations. However, nothing is known about how these variations affect their physiology. Here, by applying omics, Raman microspectroscopy, and stable isotope labeling, we investigated the effect of oxygen on “*Candidatus* Thiosymbion oneisti.” Unexpectedly, sulfur oxidation genes were upregulated in anoxic relative to oxic conditions, but carbon fixation genes and incorporation of ^13^C-labeled bicarbonate were not. Instead, several genes involved in carbon fixation were upregulated under oxic conditions, together with genes involved in organic carbon assimilation, polyhydroxyalkanoate (PHA) biosynthesis, nitrogen fixation, and urea utilization. Furthermore, in the presence of oxygen, stress-related genes were upregulated together with vitamin biosynthesis genes likely necessary to withstand oxidative stress, and the symbiont appeared to proliferate less. Based on its physiological response to oxygen, we propose that “*Ca.* T. oneisti” may exploit anaerobic sulfur oxidation coupled to denitrification to proliferate in anoxic sand. However, the ectosymbiont would still profit from the oxygen available in superficial sand, as the energy-efficient aerobic respiration would facilitate carbon and nitrogen assimilation.

**IMPORTANCE** Chemoautotrophic endosymbionts are famous for exploiting sulfur oxidization to feed marine organisms with fixed carbon. However, the physiology of thiotrophic bacteria thriving on the surface of animals (ectosymbionts) is less understood. One longstanding hypothesis posits that attachment to animals that migrate between reduced and oxic environments would boost sulfur oxidation, as the ectosymbionts would alternatively access sulfide and oxygen, the most favorable electron acceptor. Here, we investigated the effect of oxygen on the physiology of “*Candidatus* Thiosymbion oneisti,” a gammaproteobacterium which lives attached to marine nematodes inhabiting shallow-water sand. Surprisingly, sulfur oxidation genes were upregulated under anoxic relative to oxic conditions. Furthermore, under anoxia, the ectosymbiont appeared to be less stressed and to proliferate more. We propose that animal-mediated access to oxygen, rather than enhancing sulfur oxidation, would facilitate assimilation of carbon and nitrogen by the ectosymbiont.

## INTRODUCTION

At least six animal phyla and numerous lineages of bacterial symbionts belonging to *Alphaproteobacteria*, *Gammaproteobacteria*, and *Campylobacteria* (formerly *Epsilonproteobacteria*) (1) engage in chemosynthetic symbioses, rendering the evolutionary success of these associations incontestable (2, 3). Many of these mutualistic associations rely on sulfur-oxidizing (thiotrophic), chemoautotrophic bacterial symbionts that oxidize reduced sulfur compounds for energy generation in order to fix inorganic carbon (CO_2_) for biomass buildup. Particularly in binary symbioses involving thiotrophic endosymbionts, it is generally accepted that the bacterial chemosynthetic metabolism serves to provide organic carbon for feeding the animal host (reviewed in references 2 to 4). In addition, some chemosynthetic symbionts have been found capable of fixing atmospheric nitrogen, albeit symbiont-to-host transfer of fixed nitrogen remains unproven (5, 6). As for the rarer chemosynthetic bacterial-animal associations in which symbionts colonize exterior surfaces (ectosymbioses), fixation of inorganic carbon and transfer of organic carbon to the host have been unequivocally shown only for the microbial community colonizing the gill chamber of the hydrothermal vent shrimp Rimicaris exoculata (7).

The majority of thioautotrophic symbioses have been described to rely on reduced sulfur compounds as electron donors and on oxygen as terminal electron acceptor (3, 4). However, given that sulfidic and oxic zones are often spatially separated, also owing to abiotic sulfide oxidation (8, 9), chemosynthetic symbioses (i) are typically found where sulfide and oxygen occur in close proximity (e.g., hydrothermal vents, shallow-water sediments) and/or (ii) exhibit host behavioral, physiological, and anatomical adaptations enabling the symbionts to access both substrates. Among the former adaptations, host-mediated migration across oxygen and sulfide gradients was proposed for shallow-water interstitial invertebrates and *Kentrophoros* ciliates (reviewed in references 2 and 3). The ectosymbionts of Stilbonematinae, a free-living nematode subfamily of the Desmodoridae that inhabit marine sediments (2, 10), have also long been hypothesized to associate with their motile hosts to maximize sulfur oxidation-fueled chemosynthesis, by alternatively accessing oxygen in upper sand layers and sulfide in deeper, anoxic sand. This hypothesis was based upon the distribution pattern of Stilbonematinae in sediment cores together with their migration patterns observed in agar columns (10–12). However, several chemosynthetic symbionts were subsequently shown to use nitrate as an alternative electron acceptor, and nitrate respiration was stimulated by sulfide, suggesting that some may gain energy by respiring nitrate in addition to oxygen (13–17). Furthermore, although physiological studies on chemosynthetic symbioses are available (e.g., references 18 to 21), the impact of oxygen on the symbionts’ central metabolism has not been investigated (remarkably, not even in those symbionts that cover their hosts and are, therefore, directly exposed to fluctuating concentrations of oxygen).

Here, to understand how oxygen affects symbiont physiology, we focused on “*Candidatus* Thiosymbion oneisti,” a gammaproteobacterium belonging to the basal family of *Chromatiaceae* (also known as purple sulfur bacteria), which colonizes the surface of the marine nematode Laxus oneistus (Stilbonematinae). This group of free-living roundworms represents the only known animals engaging in monospecific ectosymbioses, i.e., a given nematode species is typically ensheathed by a single “*Ca.* Thiosymbion” phylotype, and in the case of “*Ca*. T. oneisti,” the bacteria form a single layer on the cuticle of their host (22–25). Moreover, the rod-shaped representatives of this bacterial genus, including “*Ca*. T. oneisti,” divide by FtsZ-based longitudinal fission, a unique reproductive strategy which ensures continuous and transgenerational host attachment (26–28).

Like other chemosynthetic symbionts, “*Ca*. Thiosymbion” bacteria have been considered chemoautotrophic sulfur oxidizers based on several lines of evidence: stable carbon isotope ratios of symbiotic nematodes are comparable to those found in other chemosynthetic symbioses (12); the key enzyme for carbon fixation via the Calvin-Benson-Bassham (CBB) cycle (RuBisCO) along with elemental sulfur and enzymes involved in sulfur oxidation have been detected (29–31); reduced sulfur compounds (sulfide, thiosulfate) have been shown to be taken up from the environment by the ectosymbionts, to be used as energy source, and to be responsible for the white appearance of the symbiotic nematodes (11, 15, 31); and the animals often occur in the sulfidic zone of marine shallow-water sands (10). More recently, the phylogenetic placement and genetic repertoire of “*Ca*. Thiosymbion” species have equally been supporting the chemosynthetic nature of the symbiosis (6, 25).

In this study, we incubated nematodes associated with “*Ca*. T. oneisti” under conditions resembling those encountered in their natural environment and subsequently examined the ectosymbiont transcriptional responses via RNA sequencing (RNA-Seq). In combination with complementary methods such as stable isotope labeling, proteomics, Raman microspectroscopy and lipidomics, we show that the ectosymbiont exhibits specific metabolic responses to oxygen. Most strikingly, sulfur oxidation but not carbon fixation was upregulated in anoxia. Such a response in their natural environment would challenge the current opinion that sulfur oxidation requires oxygen and drives carbon fixation in chemosynthetic symbioses. We finally present a metabolic scheme of a thiotrophic ectosymbiont experiencing ever-changing oxygen concentrations, in which anaerobic sulfur oxidation coupled to denitrification may represent the preferred metabolism for growth.

## RESULTS

### Hypoxic and oxic conditions induce similar expression profiles.

**FIG 1:**
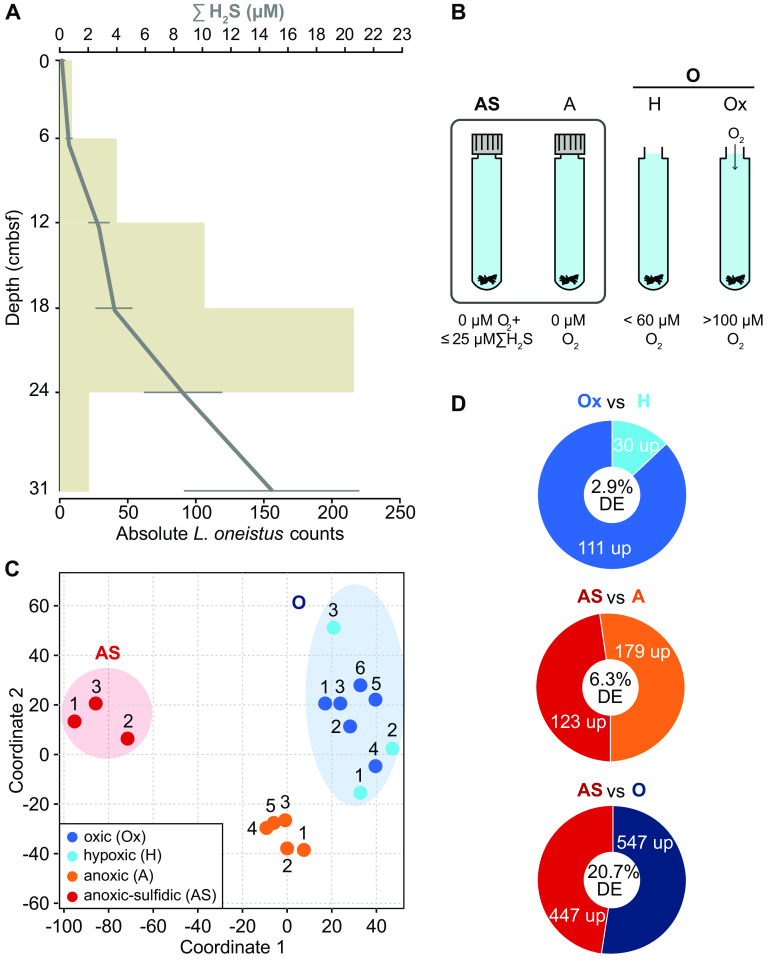
Natural and experimental conditions, transcriptome sample similarity, and differential gene expression. (A) *Laxus oneistus* total counts per 6-cm core subsection from 8 sandbars (horizontal beige bars) and corresponding mean sulfide (ΣH_2_S) concentrations (μM, gray line). Error bars represent the standard error of the mean (Table S1). (B) Experimental setup of incubations for RNA-Seq, EA-IRMS, and Raman microspectroscopy. Batches of 50 *L. oneistus* worms were incubated under different oxygen concentrations: AS (0 μM O_2_, ≤25 μM sodium sulfide added), A (0 μM O_2_), H (<60 μM O_2_ after 24 h), and Ox (>100 μM O_2_ after 24 h). The box around the anoxic incubation vials indicates that these incubations were carried out in a polyethylene isolation chamber. All incubations were performed in 0.2-μm-filtered seawater and in at least biological triplicates (see Table S2). (C) Similarity between transcriptome samples based on Euclidean distances between expression values (log_2_TPMs), visualized by means of multidimensional scaling. Most of the follow-up RNA-Seq analyses were conducted comparing the anoxic-sulfidic conditions (AS, red circle) to all conditions under which oxygen was present (O, blue circle). Samples 1 to 3 were collected in July 2017, whereas samples 4 to 6 were collected in March 2019. (D) Differential gene expression (DE) analysis between H and Ox samples revealed that the number of DE genes was low (2.9% of all expressed genes), and thus, H and Ox samples were treated as biological replicates. Of all expressed genes, 20.7% were differentially expressed between AS and O conditions. Genes were considered differentially expressed if their expression changed 2-fold with a false-discovery rate (FDR) of ≤0.05.

To understand how the movement of the animal host across the chemocline affects symbiont physiology, we exposed symbiotic worms to sulfide (thereafter used for ∑H_2_S) and oxygen concentrations resembling the ones encountered by “*Ca.* T. oneisti” in its natural habitat. Previous studies showed that Stilbonematinae live predominantly in highly reduced sediment zones with sulfide concentrations below 50 μM or up to 250 μM (10). To assess the sulfide concentration preferred by *Laxus oneistus* (i.e., the host of “*Ca.* T. oneisti”) at our collection site (Carrie Bow Cay Marine Field Station, Belize), we determined the nematode abundance relative to the sampling depth and sulfide concentration. We found the nematode abundance to be the highest between 12 and 24 cm below the seabed. Moreover, we found all *L. oneistus* individuals in pore water containing ≤25 μM sulfide. Only 1.3% of them inhabited nonsulfidic (0 μM sulfide) surface layers (Fig. 1A; see also Table S1 in the supplemental material). Therefore, we chose anoxic seawater supplemented with ≤25 μM sulfide as the incubation medium (AS condition) most resembling the natural habitat of “*Ca*. T. oneisti.” To assess the effect of oxygen on symbiont physiology, we additionally incubated the nematodes in hypoxic (H; <60 μM oxygen after 24 h) and oxic (Ox; >100 μM after 24 h) seawater (Fig. 1B). Nitrate, nitrite, ammonium, and dissolved organic carbon (DOC) could be detected throughout the sediment core including the surface layer (Table S1).

10.1128/mSystems.01186-20.7 TABLE S1 Sediment core nematode counts and chemical measurements. Download Table S1, DOCX file, 0.02 MB. Copyright © 2021 Paredes et al. 2021 Paredes et al. https://creativecommons.org/licenses/by/4.0/ This content is distributed under the terms of the Creative Commons Attribution 4.0 International license.

10.1128/mSystems.01186-20.8 TABLE S2 (A) RNA-Seq and (B) EA-IRMS incubation measurements. Replicates in bold were specifically used for Raman microspectroscopy (for further details, see Text S1) and for the assessment of the percentage of dividing cells, and thus, they were not used for EA-IRMS analysis. Each replicate consisted of a 50-worm batch. Note that RNA-Seq and EA-IRMS incubations were performed separately. Samples 1 to 3 were collected in July 2017, whereas samples 4 to 6 were collected in March 2019. T0, start of the incubation; T24, after 24 h of incubation; δ^13^C, per mille (‰). Download Table S2, DOCX file, 0.02 MB. Copyright © 2021 Paredes et al. 2021 Paredes et al. https://creativecommons.org/licenses/by/4.0/ This content is distributed under the terms of the Creative Commons Attribution 4.0 International license.

Differential gene expression analysis comparing H and Ox incubations revealed that only 2.9% of all expressed protein-coding genes differed significantly in their expression (Fig. 1C and D). Crucially, this gene set comprised several hypothetical proteins but did not show any significantly enriched metabolic pathways, processes, or protein families (Table S3B and Data Set S1). Because the presence of oxygen, irrespective of its concentration, resulted in a similar gene expression profile, we treated the samples derived from H and Ox incubations as biological replicates, and we will hereafter refer to them as the O condition.

10.1128/mSystems.01186-20.9 TABLE S3 (A) RNA sequencing and mapping statistics. Sequencing reads were mapped to the symbiont genome assembly consisting of 401 contigs and 5,169 protein-coding genes. The total number of reads refers to the number of reads after quality filtering and trimming, and the number of reads mapped to the genome (i.e., genes, intergenic regions, and antisense regions) includes only uniquely mapped reads. (B) Functional enrichments of selected gene sets. Statistical enrichment of functional categories was tested for GO terms (GO), Pfam domains (PF), KEGG metabolic maps (map), COG category (COG), and COG general category (uppercase letter) using the Bioconductor software package GOseq (M. D. Young, M. J. Wakefield, G. K. Smyth, and A. Oshlack, Genome Biol 11(2):R14, 2010, https://doi.org/10.1186/gb-2010-11-2-r14). Functional categories among sets of protein-coding genes were significantly enriched if the adjusted *P* value (false-discovery rate [FDR]) was ≤ 0.1. Only FDR values below that threshold are shown; nonsignificant FDR values are indicated (NS). AS, anoxic-sulfidic; O, hypoxic + oxic. Download Table S3, DOCX file, 0.02 MB. Copyright © 2021 Paredes et al. 2021 Paredes et al. https://creativecommons.org/licenses/by/4.0/ This content is distributed under the terms of the Creative Commons Attribution 4.0 International license.

10.1128/mSystems.01186-20.10 DATA SET S1 “*Ca*. T. oneisti” genes, functional annotations, and transcript and protein expression. Download Data Set S1, XLSX file, 2.1 MB. Copyright © 2021 Paredes et al. 2021 Paredes et al. https://creativecommons.org/licenses/by/4.0/ This content is distributed under the terms of the Creative Commons Attribution 4.0 International license.

Gene expression analysis between the AS and O conditions revealed that 20.7% of all expressed protein-coding genes exhibited significantly different expression (Fig. 1D), as we will present in detail below.

### Sulfur oxidation genes are upregulated in anoxia.

**FIG 2:**
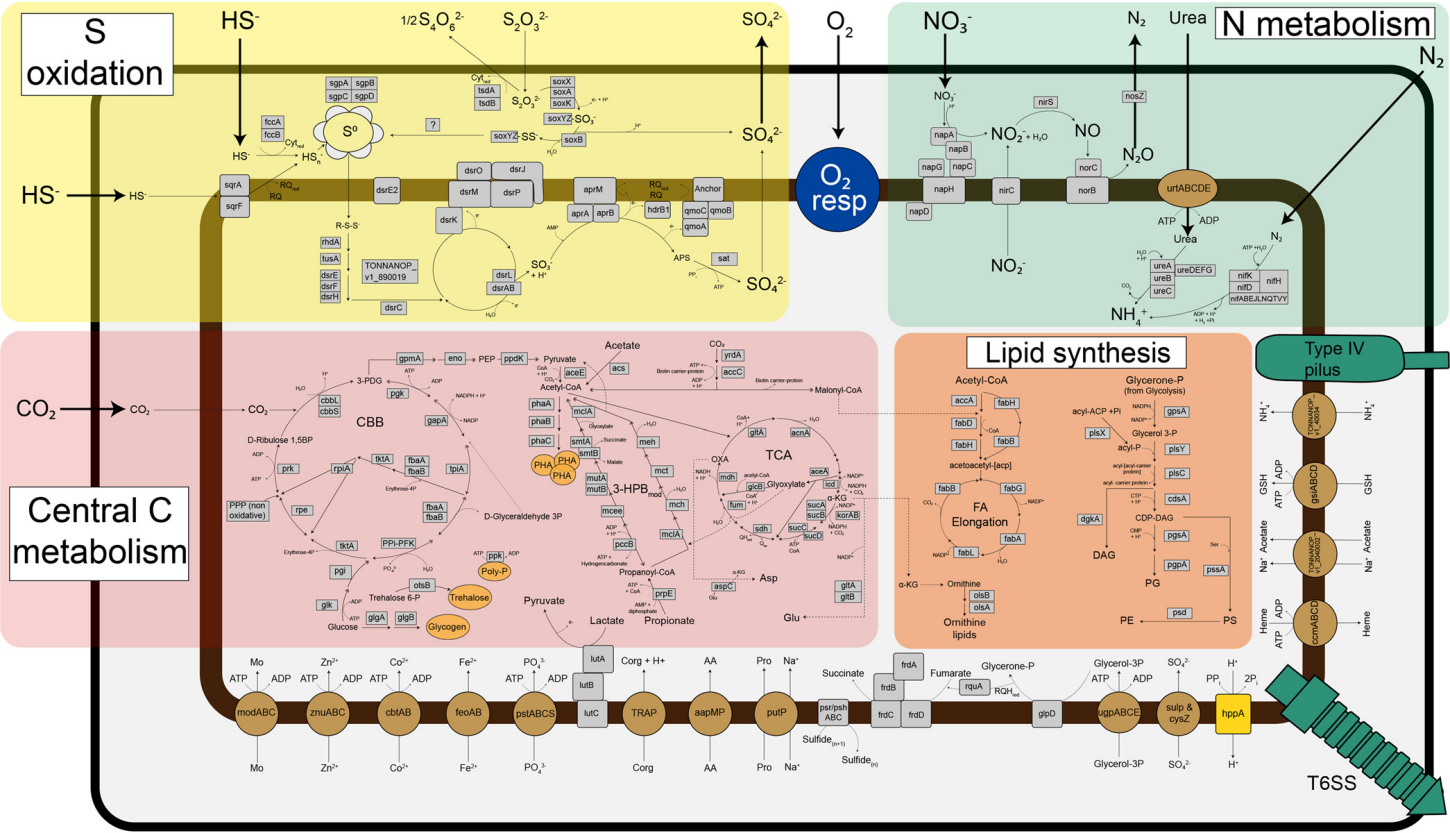
Schematic representation of central metabolic pathways present in the “*Ca*. T. oneisti” genome. All gene names (or locus tags for unidentified gene names) can be found in Data Set S1. The aerobic respiratory chain (O_2_ resp, blue) includes NADH dehydrogenase (*nuo* genes, complex I), succinate dehydrogenase (*sdh* genes, complex II), the cytochrome *bc*_1_ complex (*pet* genes, complex III), and an *aa*_3_-type cytochrome *c* oxidase (*cta* genes, complex IV). The electron transfer reactions in the S oxidation pathways are based on the work of Dahl et al. (32). Electron transfers in the denitrification pathway (N metabolism) are not illustrated but involve complexes I and III and cytochrome *c* (35). Pathways for glycogen, trehalose, and PHA degradation, as well as overall reaction stoichiometry, are not depicted. Organic carbon compounds (Corg) such as acetate, lactate, propionate, and glycerol 3-phosphate (glycerol-3P) could be host derived. Enzymes are shown in gray, transporters are brown, storage compounds are orange, and pilus and secretion system are depicted in green. AA, l-amino acids; Anchor, putative membrane anchor for the Qmo complex (TONNANOP_v1_730022); Asp, aspartate; Biotin carrier-protein, a [biotin carboxyl-carrier-protein dimer]-N6-biotinyl-l-lysine; C, carbon; CBB, Calvin-Benson-Bassham cycle; Co, cobalt; CoA, coenzyme A; DAG, diacylglycerol; FA, fatty acids; Fe, iron; Glu, glutamate; GSH, glutathione; Mo, molybdate; N, nitrogen; OXA, oxaloacetate; PE, phosphatidylethanolamine; PEP, phosphoenolpyruvate; PG, phosphatidylglycerol; PHA, polyhydroxyalkanoate; Poly-P, polyphosphate; PS, phosphatidylserine; Red, reduced; RQ, rhodoquinone; S, sulfur; Ser, serine; TCA, tricarboxylic acid cycle; TONNANOP_v1_890019, Alvin_2107 homolog (32); T6SS, type VI secretion system; Zn, zinc; 3-HPB_mod_, modified 3-hydroxypropionate cycle according to the work of Kleiner et al. (50); 3-PDG, 3-phospho-d-glycerate; α-KG, 2-oxoglutarate.

**FIG 3:**
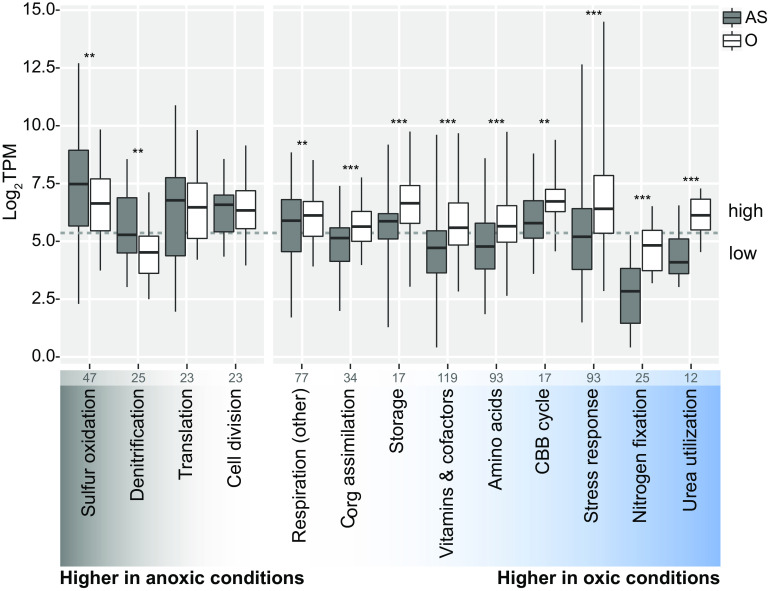
Median gene expression levels of selected “*Ca.* T. oneisti” metabolic processes under anoxic sulfidic (AS) versus oxygenated (O) conditions. All genes involved in a particular process were manually collected, and median expression levels (log_2_TPMs, transcripts per kilobase million) per condition and process are shown (horizontal bold lines). Importantly, metabolic processes include both differentially and constitutively expressed genes, and the total number of genes considered is indicated at the bottom of each process. For the specific assignment of genes, see Data Set S1. Note that for the processes designated “Amino acids,” “Storage,” and “Vitamins & cofactors,” only the expression of the biosynthesis genes was considered. Boxes indicate interquartile ranges (25% to 75%); whiskers refer to the minimum and maximum expression values, respectively. The individual processes are ordered according to the difference in median expression between AS and O conditions, i.e., sulfur oxidation (far left) had the largest difference in median expression between the two conditions, with higher median expression under the AS condition, whereas urea utilization (far right) had the largest difference in median expression, with higher median expression under the O condition. Metabolic processes were considered highly expressed when their median expression level was above 5.2 log_2_TPM (dashed gray line), which represents the median expression of all expressed protein-coding genes (*n* = 4,747) under both conditions. A Wilcoxon signed-rank test was used to test for significantly different median gene expression between conditions (**, *P* < 0.01; ***, *P* < 0.001). CBB cycle, Calvin-Benson-Bassham cycle.

**FIG 4:**
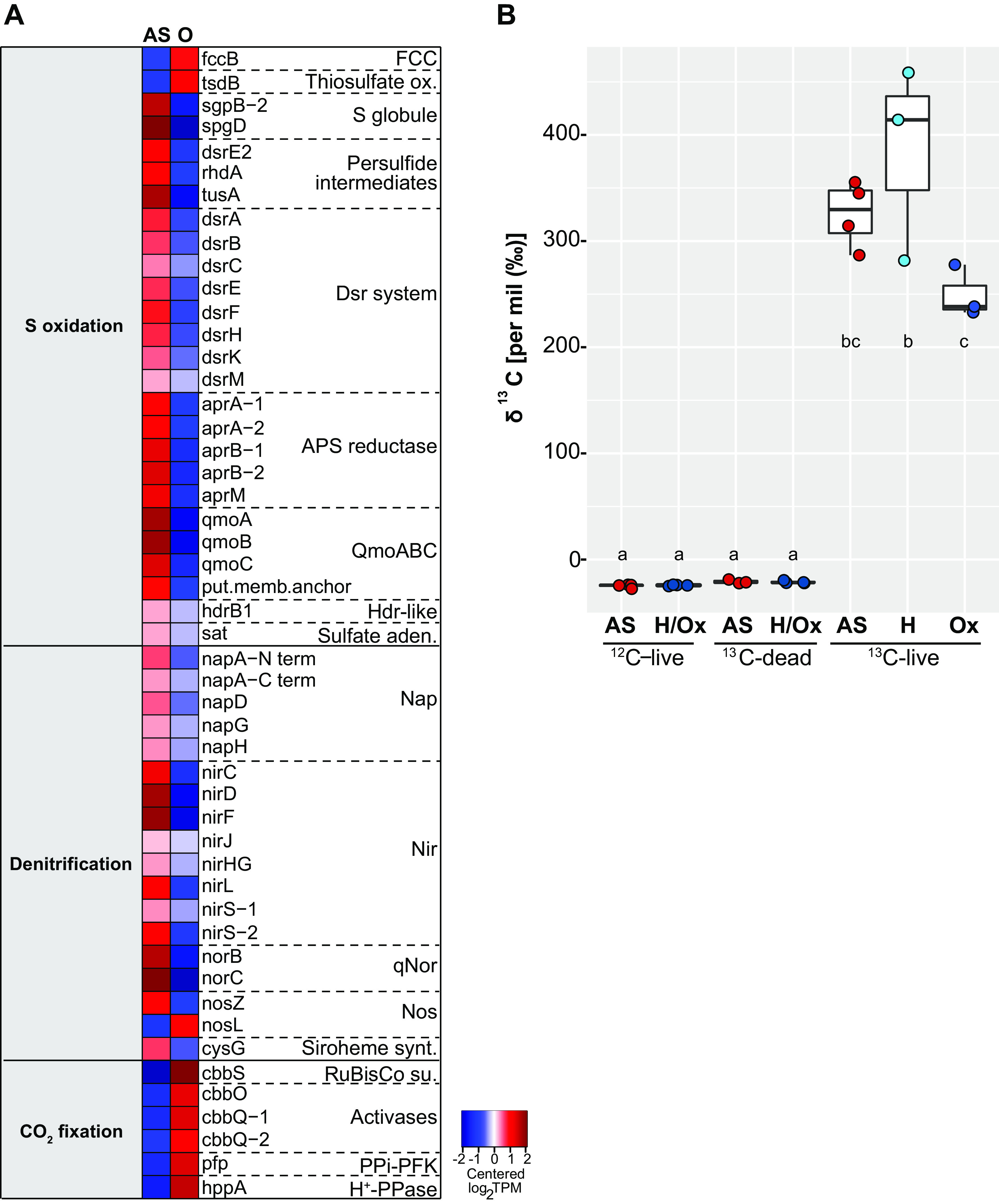
Oxidation of stored sulfur is coupled to denitrification but loosely coupled to CO_2_ fixation under anoxic conditions. (A) Heatmap visualizing only differentially expressed genes (2-fold change, FDR ≤ 0.05) involved in sulfur oxidation, denitrification, and CO_2_ fixation via the Calvin-Benson-Bassham cycle between the anoxic-sulfidic (AS) and oxygenated (O) conditions after 24 h of incubation. Expression levels are visualized by displaying mean-centered log_2_TPMs (transcripts per kilobase million). Upregulation is indicated in red, and downregulation is in blue. Genes are ordered by function in the respective metabolic pathways. FCC, flavocytochrome *c*; thiosulfate ox., thiosulfate oxidation; S, sulfur; Dsr, dissimilatory sulfite reductase; APS, adenylylsulfate; Qmo, quinone-interacting membrane-bound oxidoreductase; Hdr, heterodisulfide reductase; sat, sulfate adenylyltransferase; Nap, periplasmic nitrate reductase; Nir, cd1 nitrite reductase; qNor, quinol-dependent nitric oxide reductase; Nos, nitrous oxide reductase; Siroheme synt., siroheme biosynthesis (heme d precursor); RuBisCo su., ribulose-1,5-bisphosphate carboxylase/oxygenase small subunit; PPi-PFK, PP_i_-dependent phosphofructokinase; H^+^-PPase, proton-translocating pyrophosphatase. (B) Relative ^13^C isotope content of symbiotic *L. oneistus* as determined by EA-IRMS after 24-h incubations with ^13^C-labeled bicarbonate under anoxic-sulfidic conditions (AS, red dots), hypoxic condition (H, light blue dots), or oxic conditions (Ox, dark blue dots). Dots refer to the values determined in individual measurements (comprising 50 worms per measurement; for further details, see Table S2). Horizontal lines indicate means; error bars correspond to standard deviations. The categories ^12^C-live and ^13^C-dead refer to the natural isotope abundance control and the dead control, respectively. H/Ox, controls for both hypoxic and oxic conditions. Different lowercase letters indicate significant differences among conditions (one-way ANOVA, Tukey’s *post hoc* test, *P* < 0.05).

“*Ca.* T. oneisti” genes encoding a sulfur oxidation pathway similar to that of the related but free-living purple sulfur bacterium Allochromatium vinosum (Fig. 2) (32) were highly expressed under both AS and O conditions compared with other central metabolic processes, albeit median gene expression was significantly higher under the AS condition (Fig. 3). Consistently, 24 out of the 26 differentially expressed genes involved in sulfur oxidation were upregulated under AS (Fig. 4A). These mostly included genes involved in the cytoplasmic branch of sulfur oxidation, i.e., genes associated with sulfur transfer from sulfur storage globules (*rhd*, *tusA*, *dsrE2*), genes encoding the reverse-acting *dsr* (dissimilatory sulfite reductase) system involved in the oxidation of stored elemental sulfur (S^0^) to sulfite, and finally, also the genes required for further oxidation of sulfite to sulfate in the cytoplasm by two sets of adenylylsulfate (APS) reductase (*aprAB*) along with their membrane anchor (*aprM*) and sulfate adenylyltransferase (*sat*) (33, 34). Genes encoding a quinone-interacting membrane-bound oxidoreductase (*qmoABC*) exhibited the same expression pattern. This is noteworthy, as AprM and QmoABC are hypothesized to have an analogous function, and their cooccurrence is rare among sulfur-oxidizing bacteria (33).

Concerning genes involved in the periplasmic branch of sulfur oxidation, such as the two types of sulfide-quinone reductases (*sqrA*, s*qrF*; oxidation of sulfide) and the Sox system (*soxKAXB*, *soxYZ*) and the thiosulfate dehydrogenase (*tsdA*) both involved in the oxidation of thiosulfate, transcript levels were unchanged between the two conditions (Data Set S1). Only the flavoprotein subunit of the periplasmic flavocytochrome *c* sulfide dehydrogenase (*fccB*), as well as a cytochrome *c* family protein (*tsdB*) thought to cooperate with TsdA, was downregulated in the absence of oxygen (Fig. 4A).

To assess whether the upregulation of sulfur oxidation genes under anoxia was due to the absence of oxygen (and not to the presence of supplemented sulfide in the medium), we performed an additional anoxic incubation where sulfide was not provided (A condition). Differential expression analysis between the anoxic conditions with and without sulfide revealed that transcript levels of 6.3% of all expressed protein-coding genes differed significantly between the two anoxic treatments (Fig. 1D). Among them, we found eight genes involved in sulfur oxidation to be upregulated in the presence of sulfide (Data Set S1). Importantly, however, irrespective of sulfide supplementation, most sulfur oxidation genes were similarly upregulated in the anoxic (AS or A) relative to the O incubation (Fig. S1A, Table S3B, and Data Set S1). In addition, proteome data derived from incubations with and without oxygen, but no added sulfide, showed that one copy of AprA and one of AprM were among the top expressed proteins under anoxia (Data Set S1, column “mean %cOrgNSAF,” and Text S1). Raman microspectroscopy revealed that levels of elemental stored sulfur (S^0^) were highest under AS and H conditions and low or below detection limit under Ox conditions and A conditions at the end of the incubations (Fig. S1B and Text S1).

10.1128/mSystems.01186-20.1 TEXT S1 Description of the following methods: sediment core sampling and analysis, Raman microspectroscopy, nanometer-scale secondary ion mass spectrometry (NanoSIMS), proteomics and lipidomics, RuBisCO phylogenetic tree, and data availability for the proteomics raw data. Download Text S1, DOCX file, 0.1 MB. Copyright © 2021 Paredes et al. 2021 Paredes et al. https://creativecommons.org/licenses/by/4.0/ This content is distributed under the terms of the Creative Commons Attribution 4.0 International license.

10.1128/mSystems.01186-20.2 FIG S1 Gene expression heatmaps for sulfur oxidation and denitrification including the anoxic condition without sulfide. (A) Centered expression values (log_2_TPM) of all genes that were differentially expressed between at least two conditions are shown, with genes that were differentially expressed between the AS and O conditions, as well as the A and O conditions, in bold (twofold change, FDR ≤ 0.05). Genes are ordered by function in the respective metabolic pathways. Note that the expression of many of the genes follows a clear pattern depending on whether oxygen is present or not. (B) Relative elemental sulfur (S^0^) content in ectosymbionts as determined by Raman microspectroscopy after 24-h incubations under anoxic-sulfidic (AS, red dots), hypoxic (H, light blue dots), oxic (Ox, dark blue dots), and anoxic without added sulfide (A, orange dots) conditions. Each dot refers to the value obtained from measuring an individual ectosymbiont cell. Fifty cells were measured per condition. Horizontal lines display medians, boxes show the interquartile ranges (25 to 75%), whiskers indicate minimum and maximum values, and different lowercase letters indicate significant differences among conditions (Kruskal-Wallis test and Dunn *post hoc* test for multiple pairwise comparisons; *P* [AS versus A]] = 3.5E−17, *P* [AS versus hypoxic] = 0.011, *P* [AS versus oxic] = 7.4E−13, *P* [A versus hypoxic] = 3.2E−09, *P* [A versus oxic] = 0.188, *P* [hypoxic versus oxic] = 3.6E−06). Relative intensities below 1 (gray dashed line) indicate that elemental sulfur could not be detected. Percentage of cells with sulfur detected: 92% (AS), 16% (A), 76% (hypoxic), and 28% (oxic). For details on methodology, see Text S1. Download FIG S1, PDF file, 0.2 MB. Copyright © 2021 Paredes et al. 2021 Paredes et al. https://creativecommons.org/licenses/by/4.0/ This content is distributed under the terms of the Creative Commons Attribution 4.0 International license.

10.1128/mSystems.01186-20.4 FIG S3 NanoSIMS analysis of ^13^C isotope incorporation in *L. oneistus* and its ectosymbiont after incubation in ^13^C-labeled bicarbonate for 24 h under anoxic conditions without sulfide. The ^13^C content is displayed as ^13^C/(^12^C + ^13^C) isotope fraction, given in atom%. (A) NanoSIMS images showing cellular ultrastructure, as displayed by the ^12^C^14^N- secondary ion signal intensity (left) and isotope label distribution (right) in cross sections of *L. oneistus* after incubation of living worms in isotopically labeled (^13^C-live, top row) and unlabeled (^12^C-live, bottom row) bicarbonate. Incubation of 2% PFA-fixed worms under identical conditions in isotopically labeled bicarbonate (^13^C-dead, central row) served as a control for exclusion of unspecific (nonmetabolic) label uptake. Bars, 5 μm. (B) Region of interest (ROI)-specific evaluation of the isotopic label content, revealing significant ^13^C enrichment both in the ectosymbiont cells and, within particular regions, also in the host tissue. For details on methodology, see Text S1. Download FIG S3, PDF file, 1.3 MB. Copyright © 2021 Paredes et al. 2021 Paredes et al. https://creativecommons.org/licenses/by/4.0/ This content is distributed under the terms of the Creative Commons Attribution 4.0 International license.

Collectively, sulfur oxidation genes were upregulated under both anoxic conditions (A and AS) irrespective of sulfur storage content and, conversely, were downregulated under hypoxic conditions even though elemental sulfur was detected in most of these symbiont cells.

### Upregulation of anaerobic respiratory enzymes under AS conditions.

Given that sulfur oxidation was upregulated under AS conditions, we expected this process to be coupled to the reduction of anaerobic electron acceptors, and nitrate respiration has been shown for symbiotic *L. oneistus* (15). Consistently, genes encoding components of the four specific enzyme complexes active in denitrification (*nap*, *nir*, *nor*, *nos*), as well as two subunits of the respiratory chain complex III (*petA* and *petB* of the cytochrome *bc*_1_ complex, which is known for being involved in denitrification and in the aerobic respiratory chain [35]) were upregulated under AS conditions (Fig. 4A, Fig. S1A, and Data Set S1).

Besides nitrate respiration, “*Ca*. T. oneisti” may also utilize polysulfide or thiosulfate as a terminal electron acceptor under AS conditions, since we observed an upregulation of all genes encoding either a respiratory polysulfide reductase or a thiosulfate reductase (*psrA/phsA*, *psrB/phsB*, *prsC/phsC*; dimethyl sulfoxide [DMSO] reductase family, classification based on reference 36). Concerning other anaerobic electron acceptors, the symbiont has the genetic potential to carry out fumarate reduction (*frdABCD* genes; Fig. 2 and Data Set S1), and the fumarate reductase flavoprotein subunit (*frdA*) was indeed upregulated under AS conditions (Data Set S1). We also identified a gene potentially responsible for the biosynthesis of rhodoquinone (*rquA*; Fig. 2 and Data Set S1), which acts as an electron carrier in anaerobic respiration in a few other prokaryotic and eukaryotic organisms (37, 38) and could thus replace the missing menaquinone during anaerobic respiration in “*Ca*. T. oneisti.”

Intriguingly, lipid profiles of the symbiont revealed a change in lipid composition, as well as significantly higher relative abundances of several lysophospholipids under anoxia (Fig. S2A and Text S1), possibly resulting in altered uptake behavior and higher membrane permeability for electron donors and acceptors (39–42). Notably, we also detected lysophosphatidylcholine to be significantly more abundant in anoxia (Fig. S2B). As the symbiont does not possess any known genes for biosynthesis of this lipid, it may be host derived. Incorporation of host lipids into symbiont membranes was indeed reported previously (43, 44).

10.1128/mSystems.01186-20.3 FIG S2 Lipid composition of ectosymbionts after incubation of symbiotic nematodes under anoxic and oxic conditions. (A) Major lipid classes and their abundance relative to all lipids detected. (B) Relative abundance of significantly changed glycerophospholipids. Lipid class, fatty acid chain length, and saturation are depicted on the *x* axis. Note that PG is composed of two fatty acids, while lysophospholipids (LPG, LPE, and LPC) contain only one fatty acid. Bars show mean abundances relative to total lipids (%) and their standard deviations derived from three analytical replicates. The number of asterisks refers to the significance level (Student’s *t* test; *, *P* < 0.05; **, *P* < 0.01; ***, *P* < 0.001). Note that “*Ca*. T. oneisti” does not encode any known phosphatidylcholine biosynthesis genes. PG, phosphatidylglycerol; LPG, lysophosphatidylglycerol; LPE, lysophosphatidylethanolamine; LPC, lysophosphatidylcholine. For details on methodology see Text S1. Download FIG S2, PDF file, 0.1 MB. Copyright © 2021 Paredes et al. 2021 Paredes et al. https://creativecommons.org/licenses/by/4.0/ This content is distributed under the terms of the Creative Commons Attribution 4.0 International license.

Furthermore, upregulation of the respiratory enzyme glycerol 3-phosphate (G3P) dehydrogenase gene (*glpD*; Data Set S1), as well as the substrate-binding subunit of a putative G3P transporter gene (*ugpABCD* genes; Data Set S1), suggests that host lipid-derived G3P may serve as carbon and energy source for the symbiont under anoxia.

Taken together, our data indicate that under AS conditions, the ectosymbiont gains energy by coupling sulfur oxidation to the complete reduction of nitrate to dinitrogen gas. Moreover, the symbiont appears to exploit oxygen-depleted environments for energy generation by utilizing G3P as an additional electron donor and nitrate, polysulfide or thiosulfate, and fumarate as electron acceptors.

### Upregulation of sulfur oxidation genes is not accompanied by increased expression of carbon fixation genes.

Several thioautotrophic symbionts have been shown to use the energy generated by sulfur oxidation for the fixation of inorganic carbon (7, 19, 20, 45–47). Previous studies strongly support that “*Ca.* T. oneisti” is capable of fixing carbon via an energy-efficient Calvin-Benson-Bassham (CBB) cycle (6, 11, 12, 30, 48) (Fig. 2). In this study, bulk isotope ratio mass spectrometry (IRMS) conducted with symbiotic nematodes confirmed that they incorporate isotopically labeled inorganic carbon, and we detected no significant difference in incorporation between any two incubations in the course of 24 h (Fig. 4B). To localize the sites of carbon incorporation, we subjected symbiotic nematodes incubated with [^13^C]bicarbonate to nanoscale secondary ion mass spectrometry (NanoSIMS) and detected ^13^C enrichment predominantly within the ectosymbiont (Fig. S3 and Text S1).

Consistent with the evidence for carbon fixation by the ectosymbiont, all genes related to the CBB cycle were detected, on both the transcriptome and the proteome level, with high transcript levels under both AS and O conditions (Fig. 3 and Data Set S1). However, the upregulation of sulfur oxidation genes observed under AS did not coincide with an upregulation of carbon fixation genes. On the contrary, the median expression level of all CBB cycle genes was significantly higher in the presence of oxygen (Fig. 3). In particular, the transcripts encoding the small subunit of the key autotrophic carbon fixation enzyme ribulose-1,5-bisphosphate carboxylase/oxygenase (RuBisCO) (*cbbS*) together with the transcripts encoding its activases (*cbbQ* and *cbbO*) (49), the PP_i_-dependent 6-phosphofructokinase (PP_i_-PFK) (50, 51), and the neighboring PP_i_-energized proton pump (*hppA*) thought to be involved in energy conservation during autotrophic carbon fixation (50, 51) were upregulated under O conditions (Fig. 4A). The large subunit of the RuBisCO protein (CbbL; type I-A group according to Fig. S4) was among the top expressed proteins irrespective of the presence of oxygen (Data Set S1, column “mean %cOrgNSAF”).

10.1128/mSystems.01186-20.5 FIG S4 Phylogenetic tree of the large subunit protein of the ribulose-1,5-bisphosphate carboxylase/oxygenase (RuBisCO). Unrooted phylogenetic tree illustrating the four forms of RuBisCO from diverse organisms, such as plants and free-living and symbiotic bacteria. The CbbL protein of “*Ca*. T. oneisti” is highlighted in red. Type I (IA, IB, IC, ID), CbbL; type II, CbbM, type III; type IV, RuBisCO-like. The analysis is based on a MAFFT alignment of full-length amino acid sequences (accession numbers are provided next to the names of the organisms) and was estimated under the LG+I+G4 model using maximum likelihood phylogeny (IQ-TREE) with node support calculated by SH-aLRT. The scale bar represents 0.5% estimated sequence divergence. SH-aLRT values at the nodes are based on 10,000 replicates. For details on methodology, see Text S1. Download FIG S4, PDF file, 0.3 MB. Copyright © 2021 Paredes et al. 2021 Paredes et al. https://creativecommons.org/licenses/by/4.0/ This content is distributed under the terms of the Creative Commons Attribution 4.0 International license.

In conclusion, (i) upregulation of carbon fixation genes occurred in the presence of oxygen when sulfur oxidation genes were downregulated, while (ii) incorporation of inorganic carbon was detected to a similar extent in the presence and absence of oxygen.

### Genes involved in the utilization of organic carbon and polyhydroxyalkanoate (PHA) storage buildup are upregulated in the presence of oxygen.

As anticipated, the nematode ectosymbiont may exploit additional reduced compounds besides sulfide for energy generation. Indeed, “*Ca*. T. oneisti” possesses the genomic potential to assimilate glyoxylate, acetate, and propionate via the partial 3-hydroxypropionate cycle (like the closely related Olavius algarvensis γ1-symbiont [50]) and furthermore contains genes for utilizing additional small organic carbon compounds such as G3P, glycolate, ethanol, and lactate (Fig. 2 and Data Set S1). With the exception of G3P utilization genes (see above), the expression of genes involved in the assimilation of organic carbon including their putative transporters was significantly higher under O conditions (Fig. 3). Among the upregulated genes were *lutB* (involved in the oxidation of lactate to pyruvate [52]), propionyl coenzyme A (CoA) synthetase (*prpE*, propionate assimilation [53]), and two components of a TRAP transporter which most commonly transports carboxylates (54) (Data Set S1).

These gene expression data imply that the nematode ectosymbiont uses organic carbon compounds in addition to CO_2_ under O conditions, thereby increasing the supply of carbon. Consistent with high carbon availability, genes necessary to synthesize storage compounds such as polyhydroxyalkanoates (PHAs), glycogen, and trehalose showed an overall higher median transcript level under O conditions (Fig. 3). In particular, two key genes involved in the biosynthesis of the PHA compound polyhydroxybutyrate (PHB)—acetyl-CoA acetyltransferase (*phaA*) and a class III PHA synthase subunit (*phaC-2*)—were upregulated in the presence of oxygen. Conversely, we observed upregulation of both PHB depolymerases involved in PHB degradation under AS, and Raman microspectroscopy showed that the median PHA content was slightly lower in symbiont cells under AS than under both oxic conditions after the incubation period (Fig. S5).

10.1128/mSystems.01186-20.6 FIG S5 Differentially expressed genes involved in biosynthesis and utilization of storage compounds and detection of PHA via Raman spectroscopy. (A) Only differentially expressed genes involved in PHA and trehalose metabolism are shown (2-fold change, FDR ≤ 0.05). (B) Relative PHA content measurement of 50 ectosymbiont cells per condition after 24 h of incubation analyzed by Raman spectroscopy. Horizontal lines indicate medians, boxes show interquartile ranges (25 to 75%), and whiskers denote minimum and maximum measurements. Each dot represents a single ectosymbiont cell. PHA was detected in all cells and conditions, although more PHA was detected in cells incubated in hypoxic incubations (*P* < 0.05; Kruskal Wallis test, followed by Dunn’s multiple-pairwise-comparison test; *P* [AS versus hypoxic] = 0.022, *P* [AS versus oxic] = 0.057, *P* [hypoxic versus oxic] = 0.054). Different lowercase letters indicate significant differences among conditions. For details on methodology, see Text S1. AS, anoxic-sulfidic incubation; H, hypoxic incubation; Ox, oxic incubation. Download FIG S5, PDF file, 0.5 MB. Copyright © 2021 Paredes et al. 2021 Paredes et al. https://creativecommons.org/licenses/by/4.0/ This content is distributed under the terms of the Creative Commons Attribution 4.0 International license.

We propose that in the presence of oxygen, enhanced mixotrophy (i.e., simultaneous assimilation of inorganic and organic carbon) would result in higher carbon availability reflected by PHA storage buildup and facilitating facultative chemolithoautotrophic synthesis of ATP via the aerobic respiratory chain.

### Upregulation of nitrogen assimilation in the presence of oxygen.

**FIG 5:**
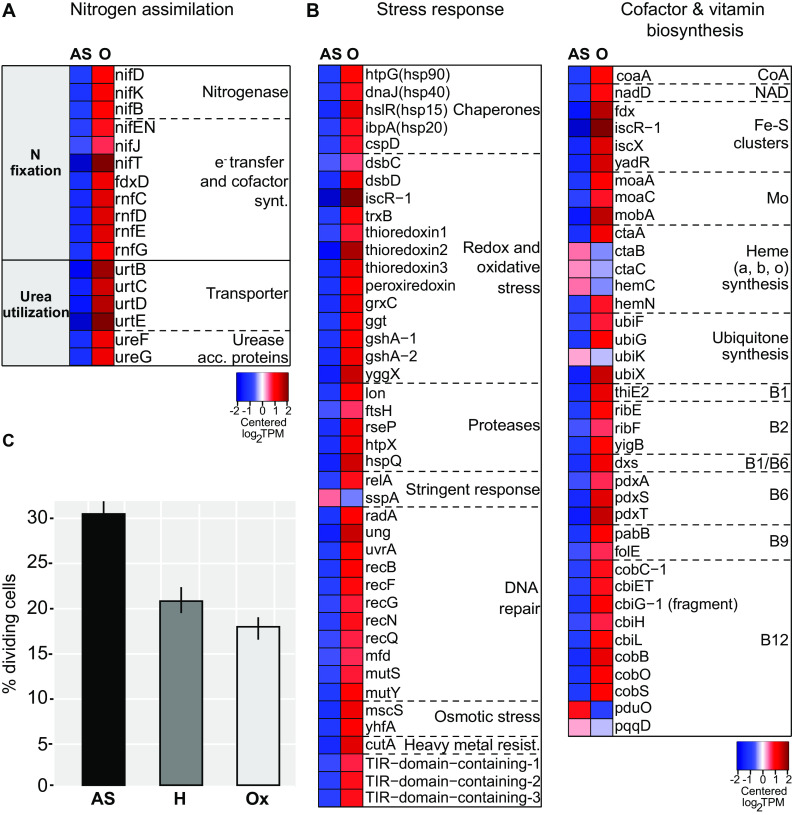
Nitrogen fixation and urea utilization genes as well as stress response and vitamin biosynthesis genes are upregulated, and fewer symbiont cells divide in the presence of oxygen. (A) Heatmap showing transcript levels of differentially expressed genes involved in nitrogen assimilation. Cofactor synt., cofactor biosynthesis; Urease acc. proteins, urease accessory proteins. (B) Heatmaps displaying transcript levels of differentially expressed genes involved in stress response as well as in the biosynthesis of vitamins and cofactors. Heavy metal resist., heavy metal resistance. Both panel A and panel B show genes that were differentially expressed between anoxic sulfidic (AS) and oxygenated (O) conditions after 24 h of incubation (2-fold change, FDR ≤ 0.05). Expression levels are visualized by displaying mean-centered log_2_TPMs (transcripts per kilobase million). Upregulation is indicated in red, and downregulation is in blue. Genes are ordered by function in the respective metabolic pathways. (C) Bars show the percentage of dividing “*Ca*. T. oneisti” cells upon 24-h incubations under anoxic sulfidic (AS), hypoxic (H), and oxic (Ox) conditions. A total of 658, 1,009, and 1,923 cells was counted for the AS, H, and Ox condition, respectively. Error bars indicate 95% confidence intervals of the proportions. A chi-square hypothesis test of independence determined that the observed differences between all proportions were highly likely dependent on the incubation condition (*P* < 0.00001).

It has been shown that high carbon availability is accompanied by high nitrogen assimilation (55–57). Indeed, despite the sensitivity of nitrogenase toward oxygen (58), its key catalytic MoFe enzymes (*nifD*, *nifK*) (59) and several other genes involved in nitrogen fixation were drastically upregulated in the presence of oxygen (Fig. 3 and 5A). Moreover, in accordance with a recent study showing the importance of sulfur assimilation for nitrogen fixation (60), genes involved in the assimilation of sulfate, i.e., the sulfate transporters *sulP* and *cys*Z, as well as genes encoding two enzymes responsible for cysteine biosynthesis (*cysM*, *cysE*) were also upregulated in the presence of oxygen (Data Set S1).

Besides nitrogen fixation, genes involved in urea uptake (transporters, *urtCBDE*) and utilization (urease, *ureF* and *ureG*) were also transcribed significantly more highly under O conditions (Fig. 3 and 5A).

In conclusion, genes involved in nitrogen assimilation (from N_2_ or urea) were consistently upregulated in the presence of oxygen, when (i) carbon assimilation was likely higher and when (ii) higher demand for nitrogen is expected due to stress-induced synthesis of vitamins (see section below).

### Upregulation of biosynthesis of cofactors and vitamins and global stress response in the presence of oxygen.

Multiple transcripts and proteins associated with diverse bacterial stress responses were among the most highly expressed in the presence of oxygen (Fig. 3 and Data Set S1). More specifically, heat shock proteins Hsp70 and Hsp90 were highly abundant (Data Set S1, column “mean %cOrgNSAF”), and transcripts of heat shock proteins (Hsp15, Hsp20, Hsp40, and Hsp90) were upregulated (Fig. 5B). Besides chaperones, we also detected upregulation of a transcription factor which induces synthesis of Fe-S clusters under oxidative stress (*iscR*) (61) along with several other genes involved in Fe-S cluster formation (Fig. 5B) (62) and regulators for redox homeostasis, like thioredoxins, glutaredoxins, and peroxiredoxins (63). Furthermore, we observed upregulation of protease genes (*lon*, *ftsH*, *rseP*, *htpX*, *hspQ*) (64–68), genes required for repair of double-strand DNA breaks (such as *radA*, *recB*, *mutSY*, and *mfd*) (69–71), and *relA*, known to initiate the stringent response when cells are starved for amino acids (72) (Fig. 5B). Amino acid starvation could be caused by a high demand for stress-related proteins under O conditions and could also explain the upregulation of amino acid biosynthesis pathways under O conditions (73) (Fig. 3).

*SspA*, shown to be important for survival under various stress conditions (74–76), was the only stress-related gene upregulated under AS (Fig. 5B).

We hypothesized that the drastic upregulation of stress-related genes observed under O conditions would require an increase in the biosynthesis of vitamins (77–79). Indeed, genes involved in biosynthesis of vitamins such as vitamins B_2_, B_6_, B_9_, and B_12_ were upregulated in the presence of oxygen (Fig. 3 and 5B). Notably, the proposed upregulation of nitrogen fixation and urea utilization (see above section) would support the synthesis of these nitrogen-rich molecules.

The upregulation of stress-related genes under O conditions was accompanied by significantly fewer dividing symbiont cells, i.e., 18.1% and 21.4% (under H and Ox conditions, respectively) versus 30.1% (under AS conditions) (Fig. 5C), and downregulation of both early (*ftsE*, *ftsX*) and late (*damX*, *ftsN*) cell division genes (80) (Fig. 3 and Data Set S1). Oxygen may therefore elicit a stress response that hampers symbiont proliferation.

## DISCUSSION

This is the first study reporting on the global transcriptional response to oxygen of a thiotrophic animal ectosymbiont, “*Ca.* T. oneisti.” Here, we detected a strong transcriptional response of “*Ca*. T. oneisti” key metabolic processes to oxygen, as well as shifts in protein abundance and lipid composition. Although ongoing comparative host transcriptomics suggests that also the nematode host responds to oxygen (L. König and G. F. Paredes, unpublished data), and although the host response likely affects that of “*Ca.* T. oneisti,” this study exclusively focused on the effect of oxygen on symbiont physiology.

### Experimental design.

The concentrations of oxygen and sulfide to which symbiotic nematodes were exposed in our study were chosen based on the distribution of *L. oneistus* and measured sulfide concentrations in their natural environment, that is, shallow-water marine sediment containing up to 25 μM sulfide (Fig. 1A), with oxidized layers rapidly transitioning to reduced, anoxic sediments (10). Given that in low-sulfide sediments, oxygen and sulfide rarely cooccur (81, 82), nematodes were not supplemented with sulfide when incubated in the presence of oxygen. Moreover, we omitted pre-experimental acclimation to study the symbiont in its close-to-natural state, i.e., replete with intracellular sulfur stores as indicated by the nematode whiteness (15, 31). Indeed, the similar gene expression observed between AS and A, and between H and O, conditions is consistent with the assumption that during the incubations, “*Ca.* T. oneisti” relied on stored sulfur, and its metabolism responded to the presence or absence of oxygen, irrespective of sulfide supplementation (Fig. 1D and Fig. 4A and see Fig. S1 in the supplemental material).

Although at the beginning of all the incubations “*Ca.* T. oneisti” was likely not depleted of stored sulfur, after 24 h of incubation, the lack of sulfide supplementation resulted in depleted sulfur stores under both the A and Ox conditions. Curiously, sulfur stores were higher following H than following Ox incubations (Fig. S1B). This cannot be explained by transcriptomics, as only a single gene involved in sulfur oxidation (a putative sulfur globule gene, *spgD* [Data Set S1]) was differentially expressed between H and Ox conditions.

Ideally, all the worms should have been subjected to the four different conditions at the same time. Although we did randomly split the worms into replicates, we were able to test a maximum of two conditions (150 nematodes per condition) per day, due to the time needed to manually extract each single nematode from the sand (see Materials and Methods). In spite of this technical limitation, replicates from the same treatment on different dates (e.g., three Ox replicates in July 2017 and three Ox replicates in March 2019) clustered with each other in their gene expression profiles (Fig. 1C).

Another potential source of variability under the conditions experienced by different nematode batches could be the fact that the A and AS conditions were tested in closed vials whereas the H and Ox ones were tested in open vials. Although in this study we measured only oxygen, sulfide, and nitrate, and we cannot, therefore, rule out whether the concentrations of other substrates differed between closed and open vials, the fact that transcriptomes of symbionts incubated in H (on the bench) and Ox (in an aquarium) samples clustered together suggests that differences in unmeasured substrates were negligible (Fig. 1C).

Overall, distinct (treatment-specific) and coherent transcriptional profiles irrespective of sampling date and experimental setup (Fig. 1C) suggest that oxygen is the main factor affecting the symbiont transcriptomes.

### Anaerobic sulfur oxidation.

**FIG 6:**
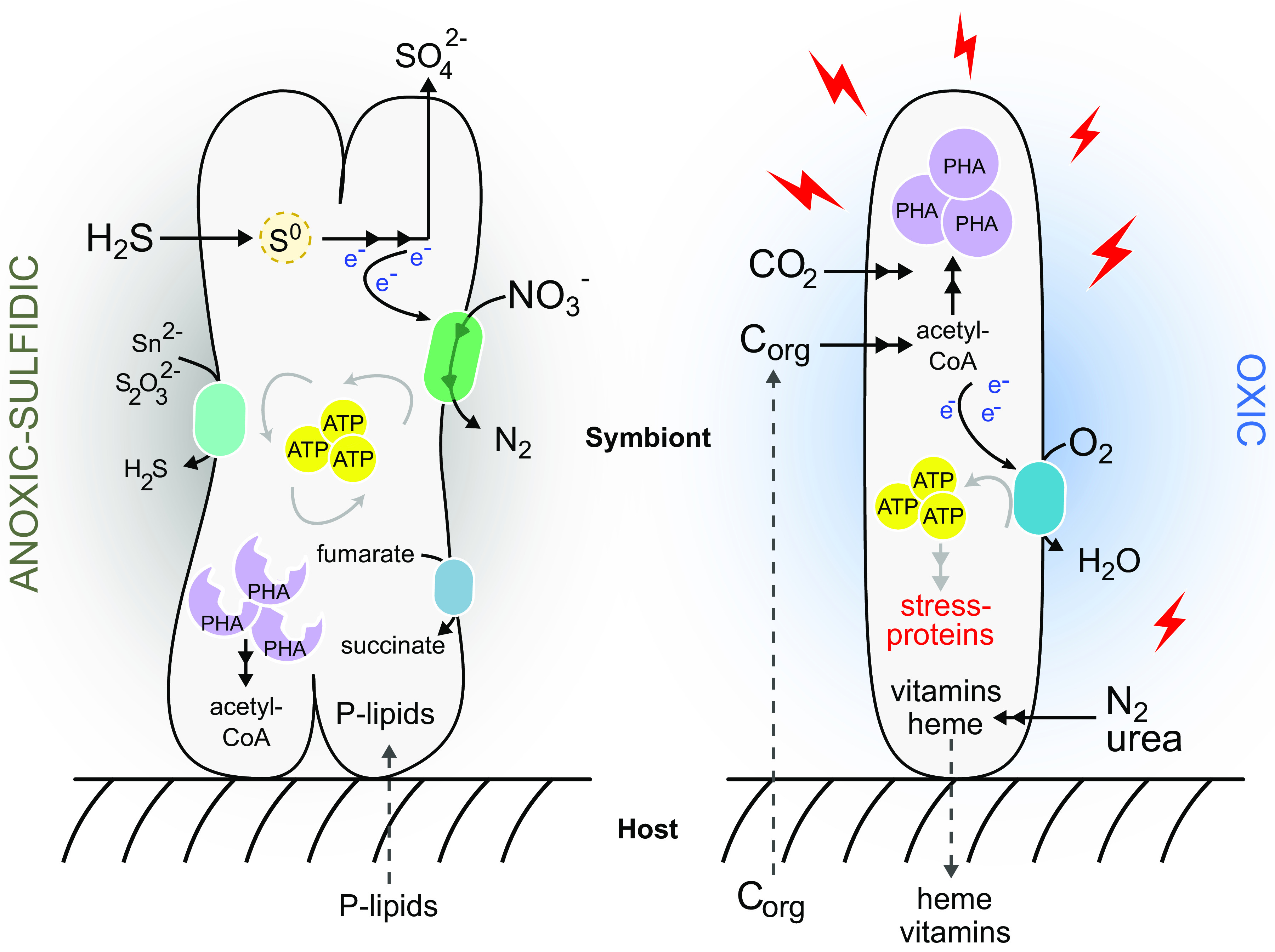
Schematic representation of the metabolism of “*Ca*. T. oneisti” in deep anoxic and upper oxygenated sand. Our study suggests that in anoxic sulfidic sediment zones (left), the ectosymbiont performs enhanced anaerobic sulfur oxidation coupled to nitrate reduction to nitrogen gas (denitrification). Additional electron acceptors such as fumarate, polysulfide (Sn^2−^), or thiosulfate (S_2_O_3_^2−^) may also be reduced. The storage compound PHA may serve as a carbon source (in addition to CO_2_) and an additional electron donor. Host-derived phospholipids (P-lipids) may be incorporated into the ectosymbiont’s membrane to increase permeability. In superficial, oxygenated zones (right), oxygen triggers a global stress response that may not only consume energy and dampen proliferation but may also require vitamin biosynthesis, thereby increasing the demand for nitrogen. Small organic carbon compounds (C_org_) putatively excreted by the host and incorporated by the ectosymbiont may contribute to energy generation (and carbon) via aerobic respiration, by the conversion of acetyl-CoA via the TCA cycle (not depicted in the figure). Together with autotrophic CO_2_ fixation, C_org_ may increase carbon availability, which would enable “*Ca.* T. oneisti” to synthesize PHA. Heme and other essential nutrients may be directly or indirectly transferred to the nematode host. Only processes predicted to dominate under one condition over the other are depicted in this model, although they likely occur under both conditions.

Genes involved in sulfur oxidation showed high overall expression compared to other central metabolic processes, indicating that thiotrophy is the predominant energy-generating process for “*Ca.* T. oneisti” under both Ox and anoxic conditions (Fig. 6). Thus, our data strongly support previous observations of Stilbonematinae ectosymbionts performing aerobic and anaerobic sulfur oxidation (11, 15). As the majority of genes involved in denitrification were upregulated under AS conditions (Fig. 4A), nitrate likely serves as terminal electron acceptor for anaerobic sulfur oxidation. Importantly, we detected nitrate in the incubation medium, as well as in all sediment layers (Table S1), at concentrations typical of oligotrophic sediment, in which also the *O. algarvensis* γ3-symbiont is predicted to couple sulfur oxidation to denitrification (50).

Sulfur oxidation in chemosynthetic symbioses is commonly described as an aerobic process required for host survival (3). However, many of these symbiotic organisms likely experience periods of oxygen depletion as would be expected from life at the interface of oxidized and reduced marine environments. Together with previous reports demonstrating nitrate reduction (13, 14, 16) and studies showing the genomic potential for using nitrate as terminal electron acceptor (6, 50, 83–85), this study substantiates that nitrate respiration during temporary anoxia could represent an important strategy for energy conservation among thiotrophic symbionts.

While upregulation of sulfur oxidation and denitrification genes in anoxia represents no proof for preferential anaerobic sulfur oxidation, we hypothesize that oxidation of reduced sulfur compounds to sulfate is more pronounced when oxygen is absent. Among the upregulated sulfur oxidation genes, we identified *aprM* and the *qmoABC* complex, both of which are thought to act as electron-accepting units for APS reductase and therefore rarely cooccur in thiotrophic bacteria (33). The presence and expression of the QmoABC complex could provide a substantial energetic advantage to the ectosymbiont by mediating electron bifurcation (33), in which the additional reduction of a low-potential electron acceptor (e.g., ferredoxin, NAD^+^) could result in optimized energy conservation under anoxic conditions. The maximization of sulfur oxidation under anoxia might even represent a temporary advantage for the host. Indeed, this would be shielded from sulfide poisoning while crawling in a sediment which is free of predators but rich in decomposed organic matter (detritus) (15, 86–88). Due to the dispensability of oxygen for sulfur oxidation, the ectosymbiont may not need to be shuttled to superficial sand by its nematode hosts to oxidize sulfur. Host migration into upper zones of the sediment may therefore primarily reflect the oxygen dependence of the animal host.

In addition to anaerobic sulfur oxidation, the nematode ectosymbiont’s phylogenetic affiliation with facultative anaerobic, anoxygenic phototrophic sulfur oxidizers such as Allochromatium vinosum (6, 32) and the presence and expression of yet other anaerobic respiratory complexes (DMSO reductase family enzyme and fumarate reductase) collectively suggest that “*Ca*. T. oneisti” might be well adapted to anoxic sulfidic sediment zones.

### Symbiont proliferation in anoxia.

Although a few studies shed light on the molecular cell biology of “*Ca*. T. oneisti” reproduction (26, 28, 89), up to this study, we did not know how this is influenced by environmental changes. Here, we observed significantly higher numbers of dividing cells under AS conditions (Fig. 5C), and therefore, sulfur oxidation coupled to denitrification might represent the ectosymbiont’s preferred strategy to generate energy for growth. We hypothesize that aside from sulfur oxidation, the mobilization of PHA could represent an additional source of ATP (and carbon) supporting symbiont proliferation under AS (Fig. S5 and Fig. 6). Of note, PHA mobilization in anoxia was also shown for *Beggiatoa* spp. (90). On the other hand, several lines of research have shown that stress—experienced by “*Ca*. T. oneisti” in the presence of oxygen (Fig. 5B)—can inhibit bacterial growth (91–97). Importantly, increased proliferation of a thiotroph which uses an anaerobic electron acceptor (such as nitrate) instead of oxygen has not been reported yet (98–101).

### Loose coupling of sulfur oxidation and carbon fixation.

Reduced sulfur compounds stimulate carbon fixation in thioautotrophic symbionts (7, 11, 19, 20, 45–47, 102, 103). Our bulk isotope ratio mass spectrometry (EA-IRMS) analysis indicates that, even though expression of the sulfur oxidation pathway was stimulated, fixation of [^13^C]bicarbonate-derived carbon was not the highest under AS conditions (Fig. 3 and 4). Instead, carbon fixation appeared unaffected by oxygen.

Even though, based on EA-IRMS, oxygen did not affect carbon fixation, CBB cycle transcripts in general, and RuBisCO-associated transcripts in particular, were significantly more abundant when oxygen was present (Fig. 3 and 4). Upregulation of these genes could be a mechanism to counteract an increased oxygenase activity of RuBisCO in the presence of oxygen, as competition between its two substrates (CO_2_ and O_2_) has been reported to constrain the carbon fixation efficiency of the enzyme (104, 105). Phylogenetic analysis of the ectosymbiont RuBisCO large subunit protein (CbbL) placed it within the type I-A group (Fig. S4), whose characterized representatives are adapted to oxic environments (105, 106). The discrepancy between carbon incorporation and transcriptome data could thus reflect a tradeoff between the carboxylase and oxygenase activity of RuBisCO. Of note, fixation of CO_2_ by other carboxylating enzymes may not significantly contribute to inorganic carbon incorporation. Indeed, acetyl-CoA carboxylase (*acc* genes) is predicted to act only as a biosynthetic carboxylase, whereas the constitutively expressed propionyl-CoA carboxylase (*pccB*) takes part in the partial 3-hydroxypropionate cycle thought to mainly function in assimilation of organic substrates in some thiotrophic symbionts (48, 50, 107). No other known carboxylases are found in the symbiont genome.

Altogether, both lines of evidence point toward a loose coupling between sulfur oxidation and autotrophic carbon fixation. Notably, sulfide oxidation without matching CO_2_ fixation has been described before for the symbiont of Riftia pachyptila (108, 109), and an example of extreme decoupling of sulfur oxidation and carbon fixation was recently reported for *Kentrophoros* ectosymbionts. Strikingly, these lack genes for autotrophic carbon fixation altogether and thus represent the first heterotrophic sulfur-oxidizing symbionts (48).

### Oxic mixotrophy.

Several chemosynthetic symbionts may engage in mixotrophy (6, 20, 50, 51, 110), and also the nematode ectosymbiont possesses genes for transport of small organic carbon compounds, their assimilation, and further metabolization (tricarboxylic acid [TCA] cycle, glyoxylate shunt). Some of the organic carbon compounds represent typical host waste products (acetate, lactate, propionate) and could therefore be host-derived (50).

The expression of genes involved in transport and assimilation pathways was significantly more pronounced under O than under AS conditions (Fig. 3). In addition to assimilating inorganic carbon autotrophically, the ectosymbiont may thus assimilate more organic carbon in the presence of oxygen and, consequently, may experience higher carbon availability (Fig. 6).

While repression of RuBisCO biosynthesis by organic carbon has been demonstrated (111, 112), simultaneous incorporation of organic and inorganic carbon has been described for several facultative autotrophic bacteria (113–119). Concomitant mixotrophy is thought to be an advantage in oligotrophic environments where nutrients are limiting (116, 120), and CO_2_ derived from the breakdown of organic carbon through decarboxylation can subsequently be reutilized via the CBB cycle (117).

The metabolization of these organic carbon compounds ultimately yields acetyl-CoA, which, in turn, can be further oxidized in the TCA cycle and/or utilized for fatty acid and PHA biosynthesis (Fig. 2 and 6). Our transcriptome and Raman microspectroscopy data suggest that “*Ca*. T. oneisti” favors PHA buildup over its degradation under O conditions (Fig. S5). Higher carbon availability in the presence of oxygen resulting in a surplus of acetyl-CoA may cause a nutrient imbalance that could facilitate PHA accumulation as previously shown (121–123). Moreover, it might play a role in resilience against cellular stress, as there is increasing evidence that PHA biosynthesis is enhanced under unfavorable growth conditions such as extreme temperatures, UV radiation, osmotic shock, and oxidative stress (124–132). Similar findings have been obtained for pathogenic (133) and symbiotic (134) bacteria of the genus *Burkholderia*, with the latter study reporting upregulation of stress response genes and PHA biosynthesis in the presence of oxygen. Finally, oxic biosynthesis of PHA might also prevent excessive accumulation and breakdown of sugars by glycolysis and oxidative phosphorylation, which, in turn, would exacerbate oxidative stress (135).

### Oxic nitrogen assimilation.

Despite the oxygen-sensitive nature of nitrogenase (58), we observed a drastic upregulation of nitrogen fixation genes under O conditions (Fig. 3 and 5A). Besides ammonia production, nitrogen fixation can act as an electron sink under heterotrophic conditions (136, 137). The ectosymbiont may therefore use the nitrogenase to maintain redox balance in the cell when organic carbon is metabolized under oxic conditions.

Urea utilization and uptake genes were also upregulated. Although the nematode host likely lacks the urea biosynthetic pathway (L. König, unpublished data), this compound is one of the most abundant organic nitrogen substrates in marine ecosystems, as well as in animal-inhabited (oxygenated) sand (138, 139). The apparent increase in nitrogen assimilation in the presence of oxygen could thus be a result of an increased demand for nitrogen driven by the biosynthesis of nitrogen-rich compounds such as vitamins and cofactors potentially required to survive oxidative stress (Fig. 3, 5, and 6). Indeed, the upregulation of the urea uptake system and urease accessory proteins, as well as the aforementioned stress-related *relA* gene, has been shown to be a response to nitrogen limitation in other systems (140, 141); nitrogen imbalance may have also induced PHA accumulation under oxic conditions (121–123). The role of vitamins in protecting cells against the deleterious effects of oxygen has been shown for animals (142, 143), and the importance of riboflavin for bacterial survival under oxidative stress has previously been reported (77, 79). Along this line of thought, oxygen-exposed “*Ca.* T. oneisti” upregulated glutathione and thioredoxin, which are known to play a pivotal role in scavenging reactive oxygen species (ROS) (144). Their function directly (or indirectly) requires vitamin B_2_, B_6_, and B_12_ as cofactors. More specifically, thioredoxin reductase (*trxB*) requires riboflavin (vitamin B_2_) in the form of flavin adenine dinucleotide (FAD) (145); cysteine synthase (*cysM*) and glutamate synthases (two-subunit *gltB*/*gltD*, one-subunit *gltS*) involved in the biosynthesis of the glutathione precursors l-cysteine and l-glutamate depend on vitamin B_6_, FAD, and riboflavin in the form of flavin mononucleotide (FMN) (146, 147). As for cobalamin, it was thought that this vitamin played only an indirect role in oxidative stress resistance (148), by being a precursor of *S*-adenosylmethionine (SAM), a substrate involved in the synthesis of glutathione via the methionine metabolism (and the transsulfuration pathway), and in preventing the Fenton reaction (149, 150). However, its direct involvement in the protection of chemolithoautotrophic bacteria against oxidative stress has also been illustrated (78).

In summary, in the presence of oxygen, the upregulation of genes involved in biosynthesis of vitamins B_2_, B_6_, and B_12_ along with antioxidant systems and their key precursor genes *cysM* and B_12_-dependent-methionine synthase *metH* suggests that the ectosymbiont requires increased levels of these vitamins to cope with oxidative stress (Fig. 6).

### Evolutionary considerations.

Anaerobic sulfur oxidation, increased symbiont proliferation, and downregulation of stress-related genes lead us to hypothesize that “*Ca.* T. oneisti” evolved from a free-living bacterium that mostly, if not exclusively, inhabited anoxic sand zones. In support of this, the closest relatives of the nematode ectosymbionts are free-living sulfur oxidizers thriving under anoxic conditions (i.e., Allochromatium vinosum, Thioflavicoccus mobilis, and Marichromatium purpuratum) (6, 151). Eventually, advantages such as protection from predators or utilization of host waste products (e.g., fermentation products, ammonia) may have been driving forces that led to the “*Ca*. Thiosymbion”-Stilbonematinae symbioses. As the association became more and more stable, the symbiont optimized (or acquired) mechanisms to resist oxidative stress, as well as metabolic pathways to most efficiently exploit the metabolic potential of oxygenated sand zones (mixotrophy, nitrogen assimilation, and vitamin and cofactor biosynthesis). From the *L. oneistus* nematode perspective, the acquired “symbiotic skin” enabled it to tolerate the otherwise poisonous sulfide and to inhabit sands virtually devoid of predators but rich in decomposed organic matter.

## MATERIALS AND METHODS

### Sample collection.

*Laxus oneistus* individuals were collected on multiple field trips (2017 to 2019) at approximately 1-m depth from a sandbar off the Smithsonian Field Station, Carrie Bow Cay, in Belize (16°48′11.01″N, 88°4′54.42″W). All the nematodes were extracted at the same location by gently stirring the sand and pouring the supernatant seawater through a 212-μm mesh sieve. The retained meiofauna was collected in a petri dish, and single worms of similar size (10 mm length, representing adult *L. oneistus*) were handpicked by using forceps (Dumont 3; Fine Science Tools, Canada) under a dissecting microscope. *L. oneistus* nematodes were identified based on morphological characteristics (152). Notably, all collected *L. oneistus* nematodes had a white appearance. Upon extraction from the sand, which required approximately 1 h per batch (50 nematodes) and 4 h for the up to 4 batches necessary to test one experimental condition (200 nematodes), the nematodes were subjected to various incubation conditions as described below.

The spatial distribution of *L. oneistus* as well as concentrations of sulfide (∑H_2_S, i.e., the sum of H_2_S, HS^−^, and S^2−^), dissolved inorganic nitrogen (DIN; nitrate, nitrite, and ammonia), and dissolved organic carbon (DOC) was determined in sediment cores at various depths (Fig. 1A; see also Table S1 and Text S1 in the supplemental material).

### Incubations for RNA sequencing (RNA-Seq).

Batches of 50 *L. oneistus* individuals were collected and incubated in triplicates or more under different oxygen concentrations during two field trips (Fig. 1B and C). Namely, they were incubated for 24 h in the dark, in either the presence or absence of oxygen, in 13-ml exetainers (Labco, Lampeter, Wales, UK) fully filled with 0.2-μm filtered seawater collected from seawater overlying the sandbar inhabited by the nematodes. The oxic incubations consisted of two separate experiments of low (hypoxic; three replicates in July 2017) and high (oxic; three replicates in July 2017, three replicates in March 2019) oxygen concentrations. Here, all exetainers were kept open, but only the samples with high oxygen concentrations were submerged in an aquarium constantly bubbled with air (Air Pump Plus; Sera, Heinsberg, Germany). Oxic incubations started with around 195 μM O_2_ and reached an average of 188 μM after 24 h. Hypoxic incubations started with around 115 μM O_2_ but reached less than 60 μM O_2_ after 24 h. This likely occurred due to nematode oxygen consumption. The anoxic treatments comprised incubations to which either 11 μM sodium sulfide (Na_2_S·9H_2_O; Sigma-Aldrich, St. Louis, MO, USA) was added (anoxic-sulfidic; three replicates in July 2017) or no sulfide was supplied (anoxic; three replicates in July 2017, two replicates in March 2019), and ∑H_2_S concentrations were checked at the beginning and at the end (24 h) of each incubation by spectrophotometric determination following the protocol of Cline (Text S1). Anoxic incubations were achieved with the aid of a polyethylene glove bag (AtmosBag; Sigma-Aldrich) that was flushed with N_2_ gas (Fabrigas, Belize City, Belize), together with incubation media and all vials, for at least 1 h before closing. Dissolved oxygen inside the bag was monitored throughout the 24 h of each incubation using a PreSens Fibox 3 trace fiber-optic oxygen meter and noninvasive trace oxygen sensor spots attached to the exetainers (PSt6 and PSt3; PreSens, Regensburg, Germany). For exact measurements of ∑H_2_S and oxygen, see Table S2A. The seawater used for all incubations had an initial concentration of nitrate and nitrite of 4.2 μM and 0.31 μM, respectively (Text S1). Temperature and salinity remained constant throughout all incubations, measuring 27 to 28°C and 33 to 34‰, respectively. All worms were moving after the 24-h incubations, indicating that they were alive. Each set of 50 worms was quickly transferred into 2 ml RNA storage solution (13.3 mM EDTA disodium dihydrate [pH 8.0], 16.6 mM sodium citrate dihydrate, 3.5 M ammonium sulfate [pH 5.2]), kept at 4°C overnight, and finally stored in liquid nitrogen until RNA extraction.

### RNA extraction, library preparation, and RNA-Seq.

RNA from symbiotic *L. oneistus* was extracted using the NucleoSpin RNA XS kit (Macherey-Nagel, Düren, Germany). Briefly, batches of 50 worms in RNA storage solution were thawed and the worms were transferred into 90 μl lysis buffer RA1 containing Tris (2-carboxyethyl) phosphine (TCEP) according to the manufacturer’s instructions. The remaining RNA storage solution was centrifuged to collect any detached bacterial cells (10 min, 4°C, 16,100 × *g*), and pellets were resuspended in 10 μl lysis buffer RA1 (plus TCEP) and then added to the worms in lysis buffer. To further disrupt cells, suspensions were vortexed for 2 min followed by three cycles of freeze (−80°C) and thaw (37°C) and homogenization using a pellet pestle (Sigma-Aldrich) for 60 s with a 15-s break after 30 s. Any remaining biological material on the pestle tips was collected by rinsing the tip with 100 μl lysis buffer RA1 (plus TCEP). Lysates were applied to NucleoSpin filters, and samples were processed according to the manufacturer’s instructions, including an on-filter DNA digest. RNA was eluted in 20 μl RNase-free water. To remove any residual DNA, a second DNase treatment was performed using the Turbo DNA-free kit (Thermo Fisher Scientific, Waltham, MA, USA), RNA was then dissolved in 17 μl RNase-free water, and the RNA quality was assessed using a Bioanalyzer (Agilent, Santa Clara, CA, USA). To check whether all DNA was digested, real-time quantitative PCR using the GoTaq qPCR master mix (Promega, Madison, WI, USA) was performed targeting a 158-bp stretch of the *sodB* gene using primers specific for the symbiont (sodB-F, GTGAAGGGTAAGGACGGTTC; sodB-R, AATCCCAGTTGACGATCTCC; 10 μM per primer). Different concentrations of genomic “*Ca.* T. oneisti” DNA were used as positive controls. The program was as follows: 1 × 95°C for 2 min, 40 × 95°C for 15 s and 60°C for 1 min, 1 × 95°C for 15 s, and 55°C to 95°C for 20 min. Next, bacterial and eukaryotic rRNA was removed using the Ribo-Zero Gold rRNA removal kit (Epidemiology) (Illumina, San Diego, CA, USA) following the manufacturer’s instructions, but volumes were adjusted for low input RNA (153). In short, 125 μl magnetic beads solution, 32.5 μl magnetic bead resuspension solution, 2 μl Ribo-Zero reaction buffer, and 4 μl Ribo-Zero removal solution were used per sample. RNA was cleaned up via ethanol precipitation and dissolved in 9 μl RNase-free water, and rRNA removal was evaluated using the Bioanalyzer RNA Pico kit (Agilent, Santa Clara, CA, USA). Strand-specific, indexed cDNA libraries were prepared using the SMARTer stranded RNA-Seq kit (TaKaRa Bio USA, Mountain View, CA, USA). Library preparation was performed according to the instructions, with 8 μl of RNA per sample as input, 3-min fragmentation time, two rounds of AMPure XP Beads (Beckman Coulter, Brea, CA, USA) cleanup before amplification, and 18 PCR cycles for library amplification. The quality of the libraries was assessed via the Bioanalyzer DNA high-sensitivity kit (Agilent). Libraries were sequenced on an Illumina HiSeq 2500 instrument (single-read, 100 nucleotides [nt]) at the next-generation sequencing facility of the Vienna BioCenter Core Facilities (VBCF; https://www.viennabiocenter.org/facilities/).

### Genome sequencing, assembly, and functional annotation.

The genome draft of “*Ca.* T. oneisti” was obtained by performing a hybrid assembly using reads from Oxford Nanopore Technologies (ONT) sequencing and Illumina sequencing. To extract DNA for ONT sequencing and dissociate the ectosymbionts from the host, approximately 800 *Laxus oneistus* individuals were incubated three times for 5 min each in TE buffer (10 mM Tris-HCl [pH 8.0], 1 mM disodium EDTA [pH 8.0]). Dissociated symbionts were collected by 10-min centrifugation at 7,000 × *g* and subsequent removal of the supernatant. DNA was extracted from this pellet using the blood and tissue kit (Qiagen, Hilden, Germany) according to the manufacturer’s instructions. The eluant was further purified using the DNA Clean & Concentrator-5 kit (Zymo Research, Irvine, CA, USA), and the DNA was eluted twice with 10 μl nuclease-free water.

The library for ONT sequencing was prepared using the ONT rapid sequencing kit (SQK-RAD002) and sequenced on an R9.4 flow cell (FLO-MIN106) on a MinION for 48 h. Basecalling was performed locally with ONT’s Metrichor Agent v1.4.2, and resulting fastq files were trimmed using Porechop v0.2.1 (https://github.com/rrwick/Porechop). Illumina sequencing reads from a previous study (6) were made available by Harald Gruber-Vodicka (MPI Bremen). Raw reads were filtered: adapters were removed and trimmed using BBDuk (BBMap v37.22, https://sourceforge.net/projects/bbmap/), with a minimum length of 36 and a minimum Phred score of 2. To keep only reads derived from the symbiont, trimmed reads were mapped onto the available genome draft (NCBI accession FLUZ00000000.1) using BWA-mem v0.7.16a-r1181 (154). Reads that did not map were discarded. The hybrid assembly was performed using SPAdes v3.11 (155) with flags –careful and the ONT reads supplied as –nanopore. Contigs smaller than 200 bp and a coverage lower than 5× were filtered out with a custom Python script. The genome completeness was assessed using CheckM v1.0.18 (156) with the gammaproteobacterial marker gene set using the taxonomy workflow. The genome was estimated to be 96.63% complete and to contain 1.12% contamination and was 4.35 Mb in length on 401 contigs with a GC content of 58.7% and *N*_50_ value of 27,060 bp.

The genome of “*Ca.* T. oneisti” was annotated using the MicroScope platform (157), which predicted 5,169 protein-coding genes. To expand the functional annotation provided by MicroScope, predicted proteins were assigned to KEGG pathway maps using BlastKOALA and KEGG Mapper-Reconstruct Pathway (158) and gene ontology (GO) terms using Blast2GO v5 (159) and searched for Pfam domains using the hmmscan algorithm of HMMER 3.0 (160, 161). All functional annotations can be found in Data Set S1. Furthermore, all genes, proteins, and pathways mentioned in the paper were manually curated and can be searched by name in Data Set S1.

### Gene expression analyses.

Based on quality assessment of raw sequencing reads using FastQC v0.11.8 (162) and prinseq-lite v0.20.4 (163), reads were trimmed and filtered using Trimmomatic v0.39 (164) and prinseq-lite as follows: 18 nucleotides were removed from the 5′ end (HEADCROP), Illumina adapters were removed (ILLUMINACLIP:TruSeq3-SE.fa:2:30:10), reads were trimmed when the average quality of a five-base sliding window dropped below a Phred score of 20 (SLIDINGWINDOW:5:20), 3′ poly(A) tails were trimmed (-trim_tail_right 1), and only reads longer than 24 nucleotides were kept (MINLEN:25). Mapping and expression analysis were done as previously described (165). Briefly, reads were mapped to the “*Ca.* T. oneisti” genome draft using BWA-backtrack (154) with default settings, only uniquely mapped reads were kept using SAMtools (166), and the number of strand-specific reads per gene was counted using HTSeq in the union mode of counting overlaps (167). On average, 1.4 × 10^6^ (4.4%) reads uniquely mapped to the “*Ca.* T. oneisti” genome. For detailed read and mapping statistics, see Table S3A.

Gene and differential expression analyses were conducted using the R software environment and the Bioconductor package edgeR v3.28.1 (168–170). Genes were considered expressed if at least two reads in at least two replicates of one of the four conditions could be assigned. Including all four conditions, we found 92.8% of all predicted symbiont protein-encoding genes to be expressed (4,797 genes out of 5,169, Data Set S1). Log_2_TPM (transcripts per kilobase million) values were calculated by log-transforming TPMs to which library size-adjusted positive prior counts were added in order to avoid zero TPMs (edgeR function addPriorCount, prior.count = 4). Log_2_TPM values were used to assess sample similarities via multidimensional scaling based on Euclidean distances (R Stats package) (170) (Fig. 1C), and the average of replicate log_2_TPM values per expressed gene and condition was used to estimate expression strength. Median gene expression of entire metabolic processes and pathways per condition was determined from average log_2_TPMs. A Wilcoxon signed-rank test was applied to test for significantly different median gene expression between metabolic processes and pathways (R Stats package).

For differential expression analysis, raw data were normalized by the trimmed mean of M-values (TMM) normalization method (edgeR function calcNormFactors) (171), and gene-specific biological variation was estimated (edgeR function estimateDisp). Differential expression was determined using the quasilikelihood F-test (edgeR functions glmQLFit and glmQLFTest) for pairwise comparisons (between all four conditions) and comparing both anoxic conditions individually against the average for both oxic conditions. Expression of genes was considered significantly different if their expression changed 2-fold between two treatments with a false-discovery rate (FDR) of ≤0.05 (172). Throughout the paper, all genes meeting these thresholds are either termed differentially expressed or up- or downregulated. However, most follow-up analyses were conducted considering only differentially expressed genes between the anoxic-sulfidic (AS) condition and the two oxygenated conditions combined (O [Results and Fig. 1C]). For the differential expression analyses between all four conditions, see Data Set S1. Heatmaps show mean-centered expression values to highlight gene expression change.

### Bulk δ^13^C isotopic analysis by Isoprime isotope ratio mass spectrometry (EA-IRMS).

To analyze the assimilation of carbon dioxide (CO_2_) by the symbionts in the presence or absence of oxygen, batches of 50 freshly collected, live worms were incubated for 24 h in 150 ml of 0.2-μm-filtered seawater, supplemented with 2 mM (final concentration) either ^12^C-labeled (natural isotope abundance control) or ^13^C-labeled sodium bicarbonate (Sigma-Aldrich, St. Louis, MO, USA). In a second control experiment, 50 freshly collected worms were killed by incubating them in a 2% paraformaldehyde/water solution for 12 h prior to 24 h of incubation with ^13^C-labeled sodium bicarbonate (dead control).

All three incubations were performed in biological triplicates or quadruplets and set up under anoxic-sulfidic and oxic conditions. Like the RNA-Seq experiment, the oxic incubations consisted of two separate experiments of low (hypoxic) and high (oxic) oxygen concentrations. To prevent isotope dilution through exchange with the atmosphere, both the oxic and anoxic incubations remained closed throughout the 24 h. The procedure was as follows: 0.2-μm-filtered anoxic seawater was prepared as described above and was subsequently used for both oxic and anoxic incubations. Then, compressed air (DAN oxygen kit; Divers Alert Network, USA) and 25 μM sodium sulfide (Na_2_S·9H_2_O; Sigma-Aldrich, St. Louis, MO, USA) were injected into the oxic and anoxic incubations, respectively, to obtain concentrations resembling the conditions applied in incubations for the RNA-Seq experiment (see Table S2B for details about the number of replicates, incubation conditions, and a compilation of the measurement data).

At the end of each incubation (24 h), the nematodes were weighed (0.3 to 0.7 mg [dry weight]) into tin capsules (Elemental Microanalysis, Devon, United Kingdom) and dried at 70°C for at least 24 h. Samples were analyzed using a Costech (Valencia, CA, USA) elemental analyzer interfaced with a continuous flow Micromass (Manchester, United Kingdom) Isoprime isotope ratio mass spectrometer (EA-IRMS) for determination of ^13^C/^12^C isotope ratios. Measurement values are displayed in δ notation (per mille [‰]). A protein hydrolysate, calibrated against NIST reference materials, was used as a standard in sample runs. The achieved precision for δ^13^C was ±0.2 ‰ (1 standard deviation of 10 replicate measurements on the standard). Statistically significant differences were determined by applying one-way analysis of variance (ANOVA), followed by Tukey’s pairwise comparisons.

### Assessment of the percentage of dividing cells.

Three individual nematodes per EA-IRMS incubation (see Table S2B for O_2_ and H_2_S measurements at the beginning and at the end of the incubations) were fixed, and ectosymbionts were dissociated from their hosts as described for Raman microspectroscopy (Text S1). A 1.5-μl amount of each bacterial suspension per condition was applied to a 1% agarose-covered slide (173), and cells were imaged using a Nikon Eclipse NI-U microscope equipped with an MFCool camera (Jenoptik). Images were obtained using the ProgRes Capture Pro 2.8.8 software (Jenoptik) and processed with ImageJ (174). Bacterial cells were manually counted (>600 per sample) and grouped into constricted (dividing) and nonconstricted (nondividing) cells based on visual inspection (28). The percentage of dividing cells was calculated by counting the total number of dividing cells and the total amount of cells per condition. The chi-square hypothesis test of independence was applied to test for a significant relationship between percentage of dividing cells and incubation condition.

### Data availability.

The assembled and annotated genome of “*Ca*. T. oneisti” has been deposited at DDBJ/ENA/GenBank under the accession no. JAAEFD000000000. RNA-Seq data are available at the Gene Expression Omnibus (GEO) database and are accessible through accession number GSE146081.
