# Supplementary material for: Anaerobic Sulfur Oxidation Underlies Adaptation of a Chemosynthetic Symbiont to Oxic-Anoxic Interfaces
Source: mSystems. 2021 May 26;6(3):e01186-20. doi: 10.1128/mSystems.01186-20 (PMC8269255; doi:10.1128/mSystems.01186-20)
Supplement: TABLE S1 [file msystems.01186-20-st001.docx]

|  | Sediment core | | | | | | | | |  |  |
| --- | --- | --- | --- | --- | --- | --- | --- | --- | --- | --- | --- |
| *L. oneistus* counts | | | | | | | | | | |  |
| Depth (cmbsf) | A | B | C | D | E | F | G | H | I | Total |  |
| 0 to 6 | 0 | 3 | 1 | 4 | 1 | 0 | 1 | 0 | 0 | 10 |  |
| 6 to 12 | 0 | 4 | 2 | 22 | 9 | 1 | 1 | 3 | 0 | 42 |  |
| 12 to 18 | 15 | 8 | 6 | 13 | 31 | 6 | 3 | 20 | 5 | 107 |  |
| 18 to 24 | 27 | 26 | 2 | 10 | 9 | 21 | 4 | 67 | 50 | 216 |  |
| 24 to 31 | ND | ND | 12 | ND | 10 | 0 | ND | ND | ND | 22 |  |
| Relative *L. oneistus* abundance (%) | | | | | | | | | | |  |
| Depth (cmbsf) | A | B | C | D | E | F | G | H | I | Mean | SE |
| 0 to 6 | 0.00 | 7.32 | 4.35 | 8.16 | 1.67 | 0.00 | 11.11 | 0.00 | 0.00 | 3.62 | 1.35 |
| 6 to 12 | 0.00 | 9.76 | 8.70 | 44.90 | 15.00 | 3.57 | 11.11 | 3.33 | 0.00 | 10.71 | 4.34 |
| 12 to 18 | 35.71 | 19.51 | 26.09 | 26.53 | 51.67 | 21.43 | 33.33 | 22.22 | 9.09 | 27.29 | 3.78 |
| 18 to 24 | 64.29 | 63.41 | 8.70 | 20.41 | 15.00 | 75.00 | 44.44 | 74.44 | 90.91 | 50.73 | 9.37 |
| 24 to 31 | ND | ND | 52.17 | ND | 16.67 | 0.00 | ND | ND | ND | 22.95 | 12.56 |
| ∑H_2_S (µM) | | | | | | | | | | |  |
| Depth (cmbsf) | A | B | C | D | E | F | G | H | I | Mean | SE |
| 0 | 0.63 | 0.00 | 0.00 | 0.85 | 0.00 | 0.00 | ND | ND | ND | 0.25 | 0.14 |
| 6 | 0.84 | 0.42 | 0.63 | 1.48 | 1.90 | 0.00 | 0.00 | 0.11 | 0.95 | 0.70 | 0.21 |
| 12 | 1.69 | 0.63 | 2.95 | 1.48 | 6.55 | 3.70 | 6.34 | 0.32 | 1.58 | 2.80 | 0.73 |
| 18 | 2.11 | 1.90 | 2.11 | 1.69 | 13.10 | 0.95 | 6.34 | 2.22 | 4.33 | 3.86 | 1.20 |
| 24 | 5.30 | 1.90 | ND | 1.90 | 22.82 | 1.80 | 14.58 | 15.11 | 6.23 | 8.70 | 2.60 |
| 31 | ND | ND | 4.75 | ND | 16.90 | 6.44 | 31.38 | ND | ND | 14.87 | 5.30 |
| Ammonium (µM) | | | | | | | | | | |  |
| Depth (cmbsf) | A | B | C | D | E | F | G | H | I | Mean | SE |
| 0 | ND | 7.75 | ND | ND | 2.7 | 18.74 | ND | ND | ND | 9.73 | 3.87 |
| 6 | ND | 5.47 | ND | ND | 12.84 | 3.64 | ND | ND | ND | 7.32 | 2.30 |
| 12 | ND | 11.56 | ND | ND | 22.54 | 8.69 | ND | ND | ND | 14.26 | 3.45 |
| 18 | ND | 10.56 | ND | ND | 21.31 | 5.69 | 23.45 | ND | ND | 15.25 | 4.26 |
| 24 | ND | 29.73 | ND | ND | 20.44 | 13.95 | 20.28 | ND | ND | 21.11 | 2.81 |
| 31 | ND | ND | ND | ND | 5.33 | 11.7 | 37.34 | ND | ND | 18.12 | 7.99 |
| Nitrate (µM) | | | | | | | | | | |  |
| Depth (cmbsf) | A | B | C | D | E | F | G | H | I | Mean | SE |
| 0 | ND | ND | ND | ND | 1.41 | 1.13 | 0.79 | ND | ND | 1.11 | 0.25 |
| 6 | ND | 0.00 | ND | ND | 0.29 | ND | 1.08 | ND | ND | 0.46 | 0.46 |
| 12 | ND | ND | ND | ND | 0.15 | ND | 0.33 | ND | ND | 0.24* | 0.06* |
| 18 | ND | ND | ND | ND | 0.00 | ND | 0.42 | ND | ND | 0.21* | 0.14* |
| 24 | ND | ND | ND | ND | 0.38 | 0.48 | 0.34 | ND | ND | 0.38 | 0.48 |
| 31 | ND | ND | ND | ND | ND | ND | 0.47 | ND | ND | 0.47* | NA |
| Nitrite (µM) | | | | | | | | | | |  |
| Depth (cmbsf) | A | B | C | D | E | F | G | H | I | Mean | SE |
| 0 | ND | ND | ND | ND | 0.08 | 0.09 | 0.07 | ND | ND | 0.08 | 0.01 |
| 6 | ND | 0.14 | ND | ND | 0.07 | ND | 0.07 | ND | ND | 0.09 | 0.02 |
| 12 | ND | ND | ND | ND | 0.03 | ND | 0.03 | ND | ND | 0.03* | 0.0* |
| 18 | ND | ND | ND | ND | 0.01 | ND | 0.02 | ND | ND | 0.015* | 0.004* |
| 24 | ND | ND | ND | ND | 0.04 | 0.18 | 0.00 | ND | ND | 0.07 | 0.05 |
| 31 | ND | ND | ND | ND | ND | ND | 0.07 | ND | ND | 0.07* | NA |
| DOC (mg/L) | | | | | | | | | | |  |
| Depth (cmbsf) | A | B | C | D | E | F | G | H | I | Mean | SE |
| 0 | ND | ND | 2.77 | 2.86 | 4.29 | 1.24 | ND | 6.52 | 2.38 | 3.34 | 0.75 |
| 6 | ND | 1.4 | 5.34 | 1.91 | 11.15 | 2.50 | ND | ND | 1.70 | 4.00 | 1.54 |
| 12 | ND | ND | 1.39 | 4.41 | 2.10 | 6.10 | ND | 1.99 | 16.44 | 5.41 | 2.32 |
| 18 | ND | 3.10 | 3.48 | 3.28 | 4.14 | ND | ND | 4.63 | 2.60 | 3.54 | 0.30 |
| 24 | ND | 5.56 | 4.75 | 4.53 | 1.42 | 9.51 | ND | 22.66 | 9.08 | 8.22 | 2.63 |
| 31 | ND | ND | 3.47 | ND | 1.78 | 20.40 | ND | ND | ND | 8.55 | 5.95 |

*: Measurements that were taken in fewer than 3 sediment cores due to technical problems and thus, means and SE were formed only to visualize the profile trends. Abbreviations: cmbsf, centimeter below seafloor; ND: not determined due to technical difficulties, SE: standard error of the mean, NA: not applicable due to just a single measurement.
