# Supplementary material for: Anaerobic Sulfur Oxidation Underlies Adaptation of a Chemosynthetic Symbiont to Oxic-Anoxic Interfaces
Source: mSystems. 2021 May 26;6(3):e01186-20. doi: 10.1128/mSystems.01186-20 (PMC8269255; doi:10.1128/mSystems.01186-20)
Supplement: TABLE S2 [file msystems.01186-20-st002.docx]

| 1. **RNA-Seq incubations** | | | | |  | | | |  |  | | |  | | |  | | |  | | |  | | | |  |  | | |  | | | |  | |  | | |  | | | | |  |
| --- | --- | --- | --- | --- | --- | --- | --- | --- | --- | --- | --- | --- | --- | --- | --- | --- | --- | --- | --- | --- | --- | --- | --- | --- | --- | --- | --- | --- | --- | --- | --- | --- | --- | --- | --- | --- | --- | --- | --- | --- | --- | --- | --- | --- |
| Sample | O_2_ (µM) | | H_2_S (µM) | |  | |  | |  | | |  | | |  | | |  | | |  | | |  |  | | | |  | | |  |  | | | | |  | | |  | |  |  |
|  | T0 h | T24 h | T0 h | T24 h |  |  | |  | | |  | | |  | | |  | | |  | | |  | | |  | |  | | |  | | | |  | |  | | |  | |  |  |  |
| anoxic-sulfidic-1 | 0.0 | 0.0 | 11.0 | 7.0 |  | |  | |  | | |  | | |  | | |  | | |  | | |  |  | | | |  | | |  |  | | | | |  | | |  | |  |  |
| anoxic-sulfidic-2 | 0.0 | 0.0 | 11.0 | 7.0 |  | |  | |  | | |  | | |  | | |  | | |  | | |  |  | | | |  | | |  |  | | | | |  | | |  | |  |  |
| anoxic-sulfidic-3 | 0.0 | 0.0 | 11.0 | 7.0 |  | |  | |  | | |  | | |  | | |  | | |  | | |  |  | | | |  | | |  |  | | | | |  | | |  | |  |  |
| anoxic-1 | 0.0 | 0.0 | 0.0 | 0.0 |  | |  | |  | | |  | | |  | | |  | | |  | | |  |  | | | |  | | |  |  | | | | |  | | |  | |  |  |
| anoxic-2 | 0.0 | 0.0 | 0.0 | 0.0 |  | |  | |  | | |  | | |  | | |  | | |  | | |  |  | | | |  | | |  |  | | | | |  | | |  | |  |  |
| anoxic-3 | 0.0 | 0.0 | 0.0 | 0.0 |  | |  | |  | | |  | | |  | | |  | | |  | | |  |  | | | |  | | |  |  | | | | |  | | |  | |  |  |
| anoxic-4 | 0.0 | 0.0 | 0.0 | 0.0 |  | |  | |  | | |  | | |  | | |  | | |  | | |  |  | | | |  | | |  |  | | | | |  | | |  | |  |  |
| anoxic-5 | 0.0 | 0.0 | 0.0 | 0.0 |  | |  | |  | | |  | | |  | | |  | | |  | | |  |  | | | |  | | |  |  | | | | |  | | |  | |  |  |
| hypoxic-1 | 111.6 | 31.4 | 0.0 | 0.0 |  | |  | |  | | |  | | |  | | |  | | |  | | |  |  | | | |  | | |  |  | | | | |  | | |  | |  |  |
| hypoxic-2 | 118.9 | 0.2 | 0.0 | 0.0 |  | |  | |  | | |  | | |  | | |  | | |  | | |  |  | | | |  | | |  |  | | | | |  | | |  | |  |  |
| hypoxic-3 | 114.8 | 17.4 | 0.0 | 0.0 |  | |  | |  | | |  | | |  | | |  | | |  | | |  |  | | | |  | | |  |  | | | | |  | | |  | |  |  |
| oxic-1 | 192.0 | 184.8 | 0.0 | 0.0 |  | |  | |  | | |  | | |  | | |  | | |  | | |  |  | | | |  | | |  |  | | | | |  | | |  | |  |  |
| oxic-2 | 196.0 | 185.0 | 0.0 | 0.0 |  | |  | |  | | |  | | |  | | |  | | |  | | |  |  | | | |  | | |  |  | | | | |  | | |  | |  |  |
| oxic-3 | 195.0 | 188.3 | 0.0 | 0.0 |  | |  | |  | | |  | | |  | | |  | | |  | | |  |  | | | |  | | |  |  | | | | |  | | |  | |  |  |
| oxic-4 | 198.3 | 192.4 | 0.0 | 0.0 |  | |  | |  | | |  | | |  | | |  | | |  | | |  |  | | | |  | | |  |  | | | | |  | | |  | |  |  |
| oxic-5 | 196.7 | 190.5 | 0.0 | 0.0 |  | |  | |  | | |  | | |  | | |  | | |  | | |  |  | | | |  | | |  |  | | | | |  | | |  | |  |  |
| oxic-6 | 196.9 | 189.7 | 0.0 | 0.0 |  | |  | |  | | |  | | |  | | |  | | |  | | |  |  | | | |  | | |  |  | | | | |  | | |  | |  |  |
|  |  |  |  |  |  | |  | |  | | |  | | |  | | |  | | |  | | |  |  | | | |  | | |  |  | | | | |  | | |  | |  |  |

| 1. **EA-IRMS incubations** | | | | |  | |  | |  | |  |  |
| --- | --- | --- | --- | --- | --- | --- | --- | --- | --- | --- | --- | --- |
| Sample | EA-IRMS Incubation | Replicates | Weight (mg) | δ13C | | O_2_ (µM) | | | | H_2_S (µM) | | |
|  |  |  |  |  |  | T0 h | | T24 h | | T0 h | | T24 h |
| anoxic-sulfidic | ^13^C live nematodes | 1 | 1.4 | 314.4 | | 0 | | 0 | | 25 | | 1.5 |
|  |  | 2 | 1.1 | 345 | | 0 | | 0 | | 25 | | 0.8 |
|  |  | 3 | 0.9 | 355.4 | | 0 | | 0 | | 25 | | 1.5 |
|  |  | 4 | 1.2 | 286.7 | | 0 | | 0 | | 25 | | 1.2 |
|  |  | **5** | n.a. | n.a. | | 0 | | 0 | | 25 | | 1.3 |
|  | ^13^C dead nematodes | 1 | 1.1 | -18.8 | | 0 | | 0 | | 25 | | 0.7 |
|  |  | 2 | 0.8 | -22.3 | | 0 | | 0 | | 25 | | 0.5 |
|  |  | 3 | 0.9 | -21.5 | | 0 | | 0 | | 25 | | 1.5 |
|  | ^12^C live nematodes | 1 | 1.1 | -23.8 | | 0 | | 0 | | 25 | | 1.3 |
|  |  | 2 | 1.2 | -24 | | 0 | | 0 | | 25 | | 1 |
|  |  | 3 | 1.2 | -24.5 | | 0 | | 0 | | 25 | | 0.7 |
|  |  | 4 | 1 | -25.8 | | 0 | | 0 | | 25 | | 0.9 |
| hypoxic | ^13^C live nematodes | 1 | 1.3 | 281.7 | | 60 | | 45 | | 0 | | 0 |
|  |  | 2 | 1.2 | 414.2 | | 60 | | 45 | | 0 | | 0 |
|  |  | 3 | 1 | 458.7 | | 59 | | 45 | | 0 | | 0 |
|  |  | **4** | n.a. | n.a. | | 60 | | 45 | | 0 | | 0 |
|  | ^13^C dead nematodes | 1 | 1.2 | -22.2 | | 59 | | 42 | | 0 | | 0 |
|  |  | 2 | 0.3 | -21.9 | | 54 | | 45 | | 0 | | 0 |
|  |  | 3 | 1.8 | -21.5 | | 61 | | 44 | | 0 | | 0 |
|  | ^12^C live nematodes | 1 | 1 | -25 | | 57 | | 50 | | 0 | | 0 |
|  |  | 2 | 1.7 | -24.1 | | 55 | | 49 | | 0 | | 0 |
|  |  | 3  4 | 1.4  1.4 | -24.4  -24.4 | | 59  59 | | 52  52 | | 0  0 | | 0  0 |
| oxic | ^13^C live nematodes | 1 | 1 | 232.9 | | 197 | | 119 | | 0 | | 0 |
|  |  | 2 | 1.1 | 277.7 | | 196 | | 109 | | 0 | | 0 |
|  |  | 3 | 1.4 | 238.3 | | 196 | | 111 | | 0 | | 0 |
|  |  | **4** | n.a. | n.a. | | 197 | | 113 | | 0 | | 0 |

n.a.: not applicable
