# Supplementary material for: Anaerobic Sulfur Oxidation Underlies Adaptation of a Chemosynthetic Symbiont to Oxic-Anoxic Interfaces
Source: mSystems. 2021 May 26;6(3):e01186-20. doi: 10.1128/mSystems.01186-20 (PMC8269255; doi:10.1128/mSystems.01186-20)
Supplement: TABLE S3 [file msystems.01186-20-st003.docx]

| 1. **RNA sequencing and mapping statistics** | | | | | | |
| --- | --- | --- | --- | --- | --- | --- |
| Samples | Total number of reads | Number of reads mapped to symbiont genome | Number of reads mapped to symbiont genes | % reads mapped to symbiont genome | % reads mapped to symbiont genes | % mapped to rRNA genes |
| oxic-1 | 39,788,652 | 935,582 | 571770 | 1.4 | 61.1 | 2.1 |
| oxic-2 | 35,270,773 | 1,308,826 | 795913 | 2.3 | 60.8 | 1.6 |
| oxic-3 | 40,645,573 | 1,140,752 | 680586 | 1.7 | 59.7 | 1.5 |
| oxic-4 | 25,823,639 | 1,422,342 | 786363 | 3.0 | 55.3 | 1.9 |
| oxic-5 | 36,002,778 | 1,401,873 | 757557 | 2.1 | 54.0 | 1.9 |
| oxic-6 | 61,503,518 | 2,065,340 | 1178912 | 1.9 | 57.1 | 2.5 |
| hypoxic-1 | 37,528,019 | 2,064,723 | 1328297 | 3.5 | 64.3 | 1.8 |
| hypoxic-2 | 36,704,147 | 2,246,543 | 1430114 | 3.9 | 63.7 | 0.5 |
| hypoxic-3 | 39,087,262 | 868,032 | 555176 | 1.4 | 64.0 | 1.0 |
| anoxic-1 | 36,848,477 | 1,755,816 | 1180245 | 3.2 | 67.2 | 0.7 |
| anoxic-2 | 36,720,206 | 1,767,472 | 1219640 | 3.3 | 69.0 | 0.8 |
| anoxic-3 | 37,438,972 | 1,757,143 | 1153214 | 3.1 | 65.6 | 1.2 |
| anoxic-4 | 57,013,563 | 1,816,539 | 971290 | 1.7 | 53.5 | 2.8 |
| anoxic-5 | 41,002,474 | 1,738,244 | 1133192 | 2.8 | 65.2 | 2.7 |
| anoxic-sulfidic-1 | 55,241,503 | 508,069 | 341801 | 0.6 | 67.3 | 2.8 |
| anoxic-sulfidic-2 | 36,482,028 | 418,210 | 281620 | 0.8 | 67.3 | 2.1 |
| anoxic-sulfidic-3 | 39,792,029 | 349,853 | 234535 | 0.6 | 67.0 | 2.5 |

| (**B) Functional enrichments of selected gene sets** | | | | | | | | | | | | |
| --- | --- | --- | --- | --- | --- | --- | --- | --- | --- | --- | --- | --- |
|  |  | FDR (threshold 0.1) | | | | | | | | | | |
|  |  | anoxic vs AS^1^ | | hypoxic vs oxic^1^ | | anoxic vs O^1^ | | AS vs O^1^ | | unchanged over 4 conditions^2^ | | |
| Category ID | Description | AS up  n = 124 | anoxic up  n = 179 | oxic up  n = 111 | hypoxic up  n =  30 | O up  n = 433 | anoxic up  n = 366 | O up  n = 548 | AS up  n = 448 | high  n = 163 | low  n = 2184 | medium  n =  1128 |
| C | Energy production and conversion | NS | NS | NS | NS | NS | **2.9E-05** | NS | **2.4E-04** | NS | NS | NS |
| O | Post-translational modification, protein turnover, and chaperones | NS | NS | NS | NS | **0.009** | NS | **0.022** | NS | **0.085** | NS | NS |
| J | Translation, ribosomal structure and biogenesis | NS | NS | NS | NS | NS | NS | NS | NS | **0.018** | NS | **3.4E-05** |
| P | Inorganic ion transport and metabolism | NS | NS | NS | NS | NS | **0.015** | NS | NS | NS | NS | NS |
| K | Transcription | NS | NS | NS | NS | NS | **0.044** | NS | NS | NS | NS | NS |
| M | Cell wall/membrane/envelope biogenesis | NS | NS | NS | NS | NS | NS | NS | NS | NS | NS | **0.001** |
| T | Signal transduction mechanisms | NS | NS | NS | NS | NS | NS | NS | NS | **0.078** | NS | NS |
| E | Amino acid transport and metabolism | NS | NS | NS | NS | NS | NS | **0.087** | NS | NS | NS | NS |
| H | Coenzyme transport and metabolism | NS | NS | NS | NS | NS | NS | **0.087** | NS | NS | NS | NS |
| map03010 | Ribosome | NS | NS | NS | NS | NS | NS | NS | NS | **0.019** | NS | **1.3E-05** |
| map00710 | Carbon fixation in photosynthetic organisms | NS | NS | NS | NS | NS | NS | NS | NS | NS | NS | **0.021** |
| map00920 | Sulfur metabolism | NS | NS | NS | NS | NS | **0.017** | NS | **0.066** | NS | NS | NS |
| map00550 | Peptidoglycan biosynthesis | NS | NS | NS | NS | NS | NS | NS | NS | NS | NS | **0.044** |
| map01230 | Biosynthesis of amino acids | NS | NS | NS | NS | NS | NS | NS | NS | NS | NS | **0.096** |
| COG1053 | Succinate dehydrogenase/fumarate reductase, flavoprotein subunit | NS | NS | NS | NS | NS | **0.023** | NS | **0.051** | NS | NS | NS |
| COG2010 | Cytochrome C, mono- and diheme variants | NS | NS | NS | NS | NS | **0.017** | NS | **0.046** | NS | NS | NS |
| COG1708 | Predicted nucleotidyltransferase | NS | NS | NS | NS | NS | **0.045** | NS | NS | NS | NS | NS |
| GO:0051912 | CoB—CoM heterodisulfide reductase activity | **0.041** | NS | NS | NS | NS | **0.021** | NS | **0.046** | NS | NS | NS |
| GO:0020037 | Heme binding | NS | NS | NS | NS | NS | **0.001** | NS | **0.046** | NS | NS | NS |
| GO:0015934 | Large ribosomal subunit | NS | NS | NS | NS | NS | NS | NS | NS | NS | NS | **0.007** |
| GO:0005840 | Ribosome | NS | NS | NS | NS | NS | NS | NS | NS | **0.053** | NS | **0.047** |
| GO:0019843 | rRNA binding | NS | NS | NS | NS | NS | NS | NS | NS | NS | NS | **9.0E-05** |
| GO:0003735 | Structural constituent of ribosome | NS | NS | NS | NS | NS | NS | NS | NS | **0.018** | NS | **9.0E-05** |
| GO:0006412 | Translation | NS | NS | NS | NS | NS | NS | NS | NS | **0.030** | NS | **9.0E-05** |
| GO:0006508 | Proteolysis | NS | NS | NS | NS | NS | NS | NS | NS | NS | NS | **0.015** |
| GO:0009055 | Electron transfer activity | NS | NS | NS | NS | NS | **0.081** | NS | NS | NS | NS | **NS** |
| PF13442 | Cytochrome C oxidase, cbb3-type, subunit III | NS | NS | NS | NS | NS | **0.024** | NS | **0.087** | NS | NS | NS |
| PF05168 | HEPN domain | NS | NS | NS | NS | NS | **0.023** | NS | **0.073** | NS | NS | NS |
| PF13609 | Gram-negative porin | NS | **0.100** | NS | NS | NS | NS | NS | NS | NS | NS | NS |

^1^ These gene sets only comprise genes that were differentially expressed between indicated conditions (FDR ≤ 0.05, fold-change of 2).

^2^ These gene sets comprise genes that were not significantly different between any of the four conditions (oxic, hypoxic, anoxic, and anoxic-sulfidic), and were furthermore, classified based on expression level (low, medium, high) over all four conditions based on hierarchical clustering with Euclidean distances.
