# Supplementary material for: Anaerobic Sulfur Oxidation Underlies Adaptation of a Chemosynthetic Symbiont to Oxic-Anoxic Interfaces
Source: mSystems. 2021 May 26;6(3):e01186-20. doi: 10.1128/mSystems.01186-20 (PMC8269255; doi:10.1128/mSystems.01186-20)
Supplement: TEXT S1 [file msystems.01186-20-s0001.docx]

**Anaerobic sulfur oxidation underlies adaptation of a chemosynthetic symbiont to oxic-anoxic interfaces**

**Supplemental Materials and Methods**

Paredes et al.

**Sediment cores analysis.** To determine the habitat and spatial distribution of *L. oneistus*, we used cores of 60 cm length and 60 mm diameter (UWITEC, Mondsee, Austria) connected to rhizon samplers of a diameter of 2.5 mm and mean pore size of 0.15 μm (Rhizosphere Research Products, Wageningen, Netherlands). This set up allowed the collection of sand and interstitial pore water (the nematode habitat) down to a depth of 30 cm. In total, nine sediment cores were collected in July 2017 at ∼1 m depth from a sand bar off Carrie Bow Cay, Belize (16°48′11.01″N, 88°4′54.42″W).

Immediately after collection, the pore-water sulfide content (∑H_2_S, i.e. the sum of H_2_S, HS^−^ and S^2−^) was determined by the methylene-blue method [1]. In short, 670 μl of a 2% zinc acetate solution was mixed with 335 μl sample and subsequently 335 μl 0.5% *N*,*N*-dimethyl-*p*-phenylenediamine and 17 μl of 10% ferrous ammonium sulfate were added and incubated for 30 min in the dark. Surface seawater was used as a blank. Absorbance was measured at 670 nm and concentrations were quantified via calibration (measurement of ∑H_2_S standard solutions in the concentration range from 0 to 0.5 mM ∑H_2_S). Samples for dissolved inorganic nitrogen (DIN: nitrate, nitrite, and ammonia) and dissolved organic carbon measurements (DOC) were stored and transported deep-frozen, and analyzed at the University of Vienna, Austria. Nitrate (NO_3_^-^) and nitrite (NO_2_^-^) concentrations were determined according to the Griess method [2] using VCl_3_ [3], whereas the concentration of ammonium (NH_4_^+^) was measured according to Solórzano [4]. For the quantification of nitrate, nitrite, and ammonia, freshly prepared KNO_3_, NaNO_2_ and NH_4_Cl solutions ranging from 0 to 100 µM were used to create standard curves, respectively. Artificial seawater served as a blank (prepared according to [5]) and all measurements were performed in technical triplicates. DOC was measured using a Shimadzu TOC-LCPH analyzer equipped with an ASI-L autosampler. After first acidifying the sample (pH 2 to 3) with hydrochloric acid, synthetic air (carbon dioxide free gas) was bubbled for 90 seconds through the sample to eliminate the inorganic carbon component. Next, the remaining total organic carbon was determined. Thereupon, 100 µL sample were injected into the combustion tube, which was filled with an oxidation platinum standard catalyst and heated to 720°C. The resulting combustion products were subsequently dehydrated, cooled and cleaned from chlorine and other halogens. Carbon dioxide was finally detected on a non-dispersive infrared (NDIR) gas analyzer. Each measurement constituted the mean from three 100 µL sample injections.

To determine the abundance of *L. oneistus*, the sand core was subdivided into 6 cm-thick layers and nematode were extracted from each sand layer by stirring the sand in seawater and pouring the supernatant through a 212 µm-mesh sieve. The retained material was transferred into a Petri dish, and single nematodes were handpicked using pipettes under a dissecting microscope. The number of *L. oneistus* nematodes and average ∑H_2_S, nitrate and nitrite concentrations are shown in Figure 1A and Table S1. All measurement data are listed in Table S1.

**Raman microspectroscopy.** Three individual nematodes per EA-IRMS incubation (see Table S2B for O_2_ and H_2_S measurements at the beginning and at the end of the incubations), and an additional incubation under anoxic conditions without supplemented sulfide (0 µM of O_2_ and H_2_S at T0 h and T24 h), were fixed and stored in 0.1 M Trump’s fixative solution (0.1 M sodium cacodylate buffer, 2.5% GA, 2% PFA, pH 7.2, 1 000 mOsm L^-1^) [6]), and washed three times for 10 min in 1x PBS (137 mM NaCl, 2.7 mM KCl, 10 mM Na_2_HPO_4_, 1.8 mM KH_2_PO_4_, pH 7.4) before their ectosymbionts were dissociated by sonication for 40 s in 10 µl 1x PBS. 1 µl of each bacterial suspension was spotted on an aluminum-coated glass slide and measured with a LabRAM HR Evolution Raman microspectroscope (Horiba, Kyoto, Japan). 50 individual single-cell spectra were measured from each sample. All spectra were aligned by the phenylalanine peak, baselined using the Sensitive Nonlinear Iterative Peak (SNIP) algorithm of the R package “Peaks” (<https://www.rdocumentation.org/packages/Peaks/versions/0.2>), and normalized by total spectrum intensity. For calculating the relative sulfur content, the average intensity value for 212-229 wavenumbers (S_8_ peak) was divided by the average intensity of the adjacent flat region of 231-248 wavenumbers. For calculating the relative polyhydroxyalkanoate (PHA) content, the average intensity value for 1 723-1 758 wavenumbers (PHA peak) was divided by the average intensity of the adjacent flat region of 1 759-1 793 wavenumbers [7]. Median relative sulfur and PHA content (shown as relative Raman intensities) were calculated treating all individual symbiont cells per condition as replicates (Figure S1B). Statistically significant differences were determined by applying the non-parametric Kruskal-Wallis test, followed by Dunn post-hoc test for multiple pairwise comparisons.

**Nanometer scale secondary ion mass spectrometry (NanoSIMS).** NanoSIMS analysis was performed to visualize and quantify the distribution and incorporation of the ^13^C label into ectosymbiont and host biomass incubated in anoxic conditions without supplemented sulfide. The experimental set up of the incubations was identical with the incubations for EA-IRMS bulk analysis (see main Material and Methods), with the difference that here, we utilized batches of 30 worms in duplicates, and one replicate of 50 worms per incubation was used for EA-IRMS to verify the incorporation of the ^13^C isotope prior to TEM/NanoSIMS sample preparation. EA-IRMS measurement values (δ13C) for the ^13^C-live, ^13^C-dead and ^12^C-live incubations were 403.9, -3.71, and -15.2 ‰, respectively. At the end of each incubation (24 h), the symbiotic nematodes were fixed and stored in 0.1 M Trump’s fixative solution [6], at 4°C until further processing.

To obtain simultaneous information on the isotopic distribution and the site of incorporation, consecutive resin sections for TEM/NanoSIMS analysis were prepared as follows: the fixed samples were washed three times with sodium cacodylate buffer (0.1 M, pH 7.2, 1 000 mOsm L^-1^), each for 10 min at room temperature (RT). Subsequently, the washing buffer was removed, and the samples were incubated in a solution of 1% osmium tetroxide for 1.5 h at RT in a shaker of low speed. Afterwards, the samples were rinsed two times with milli-Q water, each for 10 min at RT, and dehydrated stepwise by application of a concentration series of ethanol. The series consisted of 10 min incubations in 30%, 50%, 70% and 90% ethanol completed by three times 5 min incubations in 100% ethanol. Subsequently, ethanol was substituted by acetone via three times 10 min incubations in 100% acetone. Simultaneously, a fresh mixture of low viscosity resin was prepared (for 100 ml: 48 g LV resin, 8 g VH1 hardener, 44 g VH2 hardener, 2.5 g accelerator; Electron Microscopy Science). The dehydrated samples were then infiltrated stepwise by application of a resin/acetone concentration series: (i) 1:2 resin:acetone mixture for 15 min, (ii) 1:1 resin:acetone mixture for 30 min, (iii) 2:1 resin:acetone mixture for 2 h 30 min, and (iv) 100% resin for 1 h. The final step was conducted inside a vacuum desiccator. Samples were then polymerized in a laboratory oven at 60°C for 48 h. From the obtained resin blocks, thick sections (1-2 μm) were cut by a Leica Ultracut UCT microtome to assess the quality of the embedded samples and to identify appropriate regions for TEM/NanoSIMS analysis. Subsequently, consecutive sections of 70 nm (ultra-thin) and 120 nm (semi-thin) thickness were prepared using a Leica Ultracut UCT microtome and equipped with a diamond knife (Diatome, Bern, Switzerland). The ultra-thin sections (for TEM) were deposited onto previously coated (0.5% formvar solution) slot grids, and stained with 2.5% gadolinium acetate for 25 min, followed by staining with 3% lead citrate for 8 min. After each staining step, the samples were cleaned by gently dipping into milli-Q water for ten times. TEM imaging was conducted on a Zeiss Libra 120 transmission electron microscope (Carl Zeiss AG, Oberkochen, Germany). The semi-thin sections (for NanoSIMS) were deposited onto antimony-doped silicon wafer platelets (7.1 x 7.1 x 0.7 mm; Active Business Company, Brunnthal, Germany) and analyzed on a NS 50L instrument (Cameca, Gennevilliers, France).

NanoSIMS data were recorded as multilayer image stacks by sequential scanning of a finely focused Cs^+^ primary ion beam (approx. 80 nm probe size at 2 pA beam current) and simultaneous detection of negative secondary ions and secondary electrons. Recorded images had a 512 × 512 pixel resolution and a field-of-view ranging from 30 × 30 to 60 × 60 μm^2^. The mass spectrometer was tuned for a achieving a mass resolving power of > 10 000 at mass 26 to separate ^12^C^14^N^-^ secondary ions from the isobaric species ^13^C_2_^-^. Prior to data acquisition, anaysis areas were pre-conditioned *in situ* by rastering of a high intensity, defocused Cs^+^ ion beam in the following sequence of high and extreme low ion impact energies (HE / 16 keV and EXLIE / 50 eV, respectively): HE at 100 pA beam current to a fluence of 5.0E14 ions/cm^2^; EXLIE at 400 pA beam current to a fluence of 5.0E16 ions/cm^2^; HE at 100 pA to a fluence of 2.5E14 ions/cm^2^. All images were recorded at a dwell time of 7.5 – 15 ms/pixel/cycle. Secondary ion signal intensities were corrected for detector dead time and quasi-simultaneous arrival (QSA) of secondary ions, using QSA sensitivity factors (“beta” values) of 1.10 for C^−^ and 1.05 for CN^−^ ions. Image data were evaluated using the WinImage software package v2.0.8 provided by Cameca. The carbon isotope composition is displayed as ^13^C/(^12^C + ^13^C) isotope fraction, given in at%, calculated from C_2_^-^ secondary ion signal intensities via ^13^C/(^12^C + ^13^C) = ^12^C^13^C^-^/(2*^12^C^12^C^-^ +^12^C^13^C^-^). Numerical data evaluation was performed on manually defined regions of interest (ROI). Individual ROI values from samples of the ^13^C-live incubations were considered significantly enriched in ^13^C if (i) the ^13^C isotope fraction was above the 95th percent confidence interval of the corresponding ROI values determined on the negative control samples (i.e. ^12^C-live and ^13^C-dead: natural isotope abundance control and dead control, respectively) and (ii) the statistical counting error (5σ, Poisson) was smaller than the difference between the considered ROI and the mean value measured on each control.

**Preparation of *Ca.* T. oneisti pellets for proteomics.** 500 symbiotic *Laxus oneistus* were extracted from the sand as described in the main Materials & Methods, and incubated for 24 h in 13 ml of 0.2 µm filtered seawater in exetainers either in the presence of oxygen (mean concentration of dissolved oxygen at incubation start was 195.9 µM, and 183 µM after 24 h) or in anoxic conditions (O_2_ was detected neither at incubation start, nor after 24 h; no sulfide was added). After the incubations, *Ca.* T. oneisti was dissociated from the nematodes by incubating each batch of 500 nematodes in 2 ml ddH_2_O for 1 min, then transferring them to 2 ml 0.2 µM-filtered seawater for 5 min. This osmotic shock causes *Ca.* T. oneisti to detach from the nematodes and move into the seawater, which was collected with a pipette under the dissecting microscope to exclude involuntary aspiration of nematode tissue (or fragments thereof). The 2 ml nematode-free, ectosymbiont suspension was then centrifuged for 1 min at 14 000 x g to obtain *Ca.* T. oneisti pellets. Ectosymbiont pellets and aposymbiotic nematodes were flash-frozen in liquid nitrogen and stored at ‑80°C until further processing. Only *Ca*. T. oneisti proteomic data are shown in this study. *L. oneistus* proteomics will be published separately.

**Protein extraction and 1D PAGE.** *Ca.* T. oneisti proteins were extracted as described previously [8]. Briefly, both samples, i.e. frozen ectosymbiont cell pellets from oxic and anoxic incubations, were resuspended in 1% (w/v) sodium deoxycholate (SDC), 4% (w/v) sodium dodecyl sulfate (SDS) in 50 mM triethylammonium bicarbonate buffer (lysis buffer). After boiling the samples for 5 min under agitation (600 rpm), they were incubated in an ultrasonic bath for 5 min at RT. After removal of cell debris by a 10 min centrifugation at RT (14 000 x g), protein concentrations in the supernatants were determined using the Pierce BCA (bicinchoninic acid) assay (Thermo Scientific Pierce, Waltham, MA, USA) according to the manufacturer’s instructions in a Tecan microtiter plate reader. For gel-based proteomic analysis (as previously described by [9]), 25 µg of protein per sample were mixed with loading buffer (2 % (w/v) SDS, 10 % glycerol, 12.5 mM dithiothreitol, 0.001 % (w/v) bromophenol blue in 0.06 M Tris-HCl) and separated in precast 4 – 20 % SDS mini gels (BioRad TGX). Per sample, three replicates (3 x 25 µg protein) were separated (giving a total of 6 samples). After staining with Coomassie Brilliant Blue, protein-containing gel lanes were excised and subdivided into 10 equal-sized pieces each, which were destained at 37 °C in 200 mM NH_4_HCO_3_ 30 % acetonitrile under agitation at 600 rpm and digested overnight at 37 °C with trypsin (sequencing grade; Promega, Madison, WI, USA). Finally, peptides were eluted in an ultrasonic bath and subjected to LC-MS/MS analysis.

**LC-MS/MS analysis.** Peptides were analyzed by reversed phase liquid chromatography (LC) electrospray ionization (ESI) MS/MS using an LTQ Orbitrap Velos (Thermo Fisher Scientific, Waltham, MA, USA) according to [10]. Briefly, in-house self-packed nano-LC columns (100 µm x 20 cm) containing reverse-phase C18 material (3 µm, ReproSil-Pur 120-AQ; Dr. Maisch GmbH, Ammerbuch-Entringen, Germany) were used to perform LC with an Easy-nLC1000 system (Thermo Fisher Scientific). The peptides were loaded with solvent A (0.1% acetic acid (v/v)). Subsequently, the peptides were eluted by a non-linear binary gradient of 80 minutes from 5% to 99% solvent B (0.1% acetic acid (v/v), 99.9% acetonitrile (v/v)) in solvent A at a constant flow rate of 300 nl/min. MS data were acquired in data-dependent MS/MS mode for the 20 most abundant precursor ions. After a full scan in the Orbitrap (*m/z* 300 – 1 700) with a resolution of 30 000 at *m/z* 400, ions were fragmented via collision-induced dissociation (CID) and recorded in the linear trap quadrupole LTQ analyzer.

**Protein identification and quantification***.* For protein identification, a database was constructed, containing 18 364 *Laxus oneistus* host protein sequences (derived from a *de novo* assembled transcriptome; will be published separately), 5 169 *Ca*. T. oneisti protein sequences (JAAEFD000000000, see main Materials and Methods) and a set of 42 common laboratory contaminants. All sequences were reversed and appended to the database as decoys to allow for false-discovery rate (FDR) assessment. Mass spectra were searched against this target-decoy database using the Sorcerer SEQUEST algorithm (Sage-N Research) and filtered using Scaffold (version 4.8.4, http://www.proteomesoftware.com) applying the following thresholds: i) protein FDR and peptide FDR were set to 1% and ii) at least two unique peptides were required for a protein or protein group to be identified. Proteins were expressed if they were detected in at least two out of the three replicates in at least one condition. This way, 1 137 ectosymbiont proteins (22.0% of all predicted proteins in the database) were identified in total. Data S1 indicates all detected proteins in the column “Proteome detection” (Column AB). Relative abundance of identified proteins was calculated from total spectrum counts as normalized spectral abundance factor (%NSAF) values – giving the percentage of each protein relative to all proteins in the respective sample [11], and as %OrgNSAF, giving a protein’s percentage relative to all ectosymbiont proteins in the respective sample [12]. As ectosymbiont protein identification rates varied substantially between oxic and anoxic samples, which may negatively affect comparability of relative abundances between samples, we included only such proteins in the final quantitation, which were detected under both conditions (824 proteins). This additional normalization step provided corrected %OrgNSAF values (%cOrgNSAF), which give a protein’s percentage relative to all symbiont proteins that were expressed under both conditions. %cOrgNSAF values are listed in Data S1 (columns AC and AD), and values are highlighted in yellow when the respective proteins were among the top 30 most abundant proteins.

**Intact polar lipid extraction and analysis.** Five batches of 100 freshly collected *Laxus oneistus* were incubated for 24 h in oxic or anoxic (no sulfide added) conditions as described in the main Materials and Methods (RNA-Seq incubations). At the beginning of the incubations, mean concentrations of dissolved oxygen in the 0.2 µm filtered seawater were 180.9 µM (oxic) and 0.47 µM (anoxic). After 24 h, we measured on average 86 µM (oxic) and 0 µM (anoxic) oxygen, respectively. Sulfide (∑H_2_S) could not be detected in any of the incubations. At the end of the incubations, *Ca*. T. oneisti (from either the oxic or anoxic conditions) were dissociated from 500 nematodes, as described above (Proteomics). Symbiont pellets were flash-frozen in liquid nitrogen and stored at ‑80°C until further processing.

Lipids of the ectosymbionts were extracted using a modified Folch extraction [13] previously applied for lipid extraction from bacteria [14]. Briefly, pelleted bacteria were taken up in 1.6 ml 0.2 µm filtered seawater and 0.5 ml were transferred to 2 ml glass vials obtaining three analytical replicates. Bacteria were then pelleted by centrifugation, resuspended in 0.5 ml methanol and extracted using chloroform-methanol (all solvents LC-MS grade, Sigma-Aldrich, St. Louis, MS, USA). Extracted lipids were dried under nitrogen gas on a Techne Sample Concentrator and re-suspended in 1 ml of acetonitrile: 10 mM ammonium acetate at a 95:5 (v:v) ratio. Samples were analyzed by liquid chromatography mass spectrometry (LC-MS) as follows: lipids were separated on a Dionex UltiMate 3000RS UHPLC (Thermo Fisher Scientific) equipped with a XBridge BEH amide XP column (Waters, Milford, MA, USA) and coupled to an amaZon SL quadrupole ion trap MS (Bruker, Billerica, MA, USA) for detection. The column was maintained at 30°C with a flow rate of 150 µl min^-1^. Samples were separated by a 15 min gradient from 95% (v:v) acetonitrile (Solvent A) to 30% (w:v) 10 mM ammonium acetate (pH 9.2, Solvent B) with 10 min equilibration between samples. Sample analysis was carried out in both positive and negative ion mode and fragmentation performed by the autoMS^n^ function in Compass HyStar (Bruker, Bremen, Germany). We used the Bruker Compass software package for lipid data analysis: DataAnalysis for peak detection and lipid identification, and QuantAnalysis for quantification of the relative abundances of lipids. Peak integration was manually corrected where necessary. Consecutively, for data normalization the peak area of each lipid was divided by the sum of the peak areas of all detected lipids in each sample. Statistical analysis of significant differences in ectosymbiont lipids between the anoxic and oxic condition was carried out using a Student’s t-test.

**RuBisCO phylogenetic tree**. Amino acid sequences of the RuBisCO forms I-IV were obtained from GenBank and SwissProt databases, and aligned using mafft v7.397 [15]. Please note that the accession numbers are provided next to the names of the organisms (Figure S4). Misaligned sequences were manually inspected. Gaps in more than 70% of the sequences were removed using TrimAl 1.4.rev15 [16]. The maximum-likelihood tree with SH-aLRT support values (10 000 replicates) was inferred using IQ-TREE v1.6.2 with automatic model selection [17, 18].

**Data availability**. The proteomics raw data and the combined *L. oneistus* host and ectosymbiont database used for proteomic analyses have been deposited to the ProteomeXchange Consortium via the PRIDE [19] partner repository with the data set identifier PXD017709.

**Supplemental References**

1. Cline JD. Spectrophotometric determination of hydrogen sulfide in natural waters. *Limnol Oceanogr* 1969; **14**: 454–458.

2. Green LC, Wagner DA, Glogowski J, Skipper PL, Wishnok JS, Tannenbaum SR. Analysis of nitrate, nitrite, and [15N]nitrate in biological fluids. *Anal Biochem* 1982; **126**: 131–138.

3. Schnetger B, Lehners C. Determination of nitrate plus nitrite in small volume marine water samples using vanadium(III)chloride as a reduction agent. *Mar Chem* 2014; **160**: 91–98.

4. Solórzano L. Determination of ammonia in natural waters by the phenolhypochlorite method. *Limnol Oceanogr* 1969; **14**: 799–801.

5. Kester DR, Duedall IW, Connors DN, Pytkowicz RM. Preparation of artificial seawater. *Limnol Oceanogr* 1967; **12**: 176–179.

6. Trump BF, Ericsson JL. The effect of the fixative solution on the ultrastructure of cells and tissues. A comparative analysis with particular attention to the proximal convoluted tubule of the rat kidney. *Lab Investig* 1965; **14**: 1245–1323.

7. Samek O, Obruča S, Šiler M, Sedláček P, Benešová P, Kučera D, et al. Quantitative Raman spectroscopy analysis of polyhydroxyalkanoates produced by *Cupriavidus necator* H16. *Sensors* 2016; **16**:1808.

8. Hinzke T, Markert S. Efficient protein extraction for proteomics and metaproteomics (also suitable for low biomass samples). *Protocols.io.* 2017; **10**, kg6ctze.

9. Ponnudurai R, Kleiner M, Sayavedra L, Petersen JM, Moche M, Otto A, et al. Metabolic and physiological interdependencies in the *Bathymodiolus azoricus* symbiosis. *ISME J* 2017; **11**: 463–477.

10. Chambers MC, MacLean B, Burke R, Amodei D, Ruderman DL, Neumann S, et al. A cross-platform toolkit for mass spectrometry and proteomics. *Nat Biotechnol* 2012; **30**: 918–920.

11. Florens L, Carozza MJ, Swanson SK, Fournier M, Coleman MK, Workman JL, et al. Analyzing chromatin remodeling complexes using shotgun proteomics and normalized spectral abundance factors. *Methods* 2006; **40**: 303–311.

12. Mueller RS, Denef VJ, Kalnejais LH, Suttle KB, Thomas BC, Wilmes P, et al. Ecological distribution and population physiology defined by proteomics in a natural microbial community. *Mol Syst Biol* 2010; **6**.

13. Folch J, Lees M, Sloane Stanley GH. A simple method for the isolation and purification of total lipides from animal tissues. *J Biol Chem* 1957; **226**: 497–509.

14. Smith AF, Rihtman B, Stirrup R, Silvano E, Mausz MA, Scanlan DJ, et al. Elucidation of glutamine lipid biosynthesis in marine bacteria reveals its importance under phosphorus deplete growth in Rhodobacteraceae. *ISME J* 2019; **13**: 39–49.

15. Katoh K, Standley DM. MAFFT Multiple Sequence Alignment Software Version 7: Improvements in Performance and Usability. *Mol Biol Evol* 2013; **30**: 772–780.

16. Capella-Gutiérrez S, Silla-Martínez JM, Gabaldón T. trimAl: a tool for automated alignment trimming in large-scale phylogenetic analyses. *Bioinformatics* 2009; **25**: 1972–3.

17. Nguyen L-T, Schmidt HA, von Haeseler A, Minh BQ. IQ-TREE: A Fast and Effective Stochastic Algorithm for Estimating Maximum-Likelihood Phylogenies. *Mol Biol Evol* 2015; **32**: 268–274.

18. Kalyaanamoorthy S, Minh BQ, Wong TKF, Von Haeseler A, Jermiin LS. ModelFinder: Fast model selection for accurate phylogenetic estimates. *Nat Methods* 2017; **14**: 587–589.

19. Vizcaíno JA, Côté RG, Csordas A, Dianes JA, Fabregat A, Foster JM, et al. The Proteomics Identifications (PRIDE) database and associated tools: Status in 2013. *Nucleic Acids Res* 2013; **41**.
