# Supplementary figures and images for: Anaerobic Sulfur Oxidation Underlies Adaptation of a Chemosynthetic Symbiont to Oxic-Anoxic Interfaces
Source: mSystems. 2021 May 26;6(3):e01186-20. doi: 10.1128/mSystems.01186-20 (PMC8269255; doi:10.1128/mSystems.01186-20)

**A**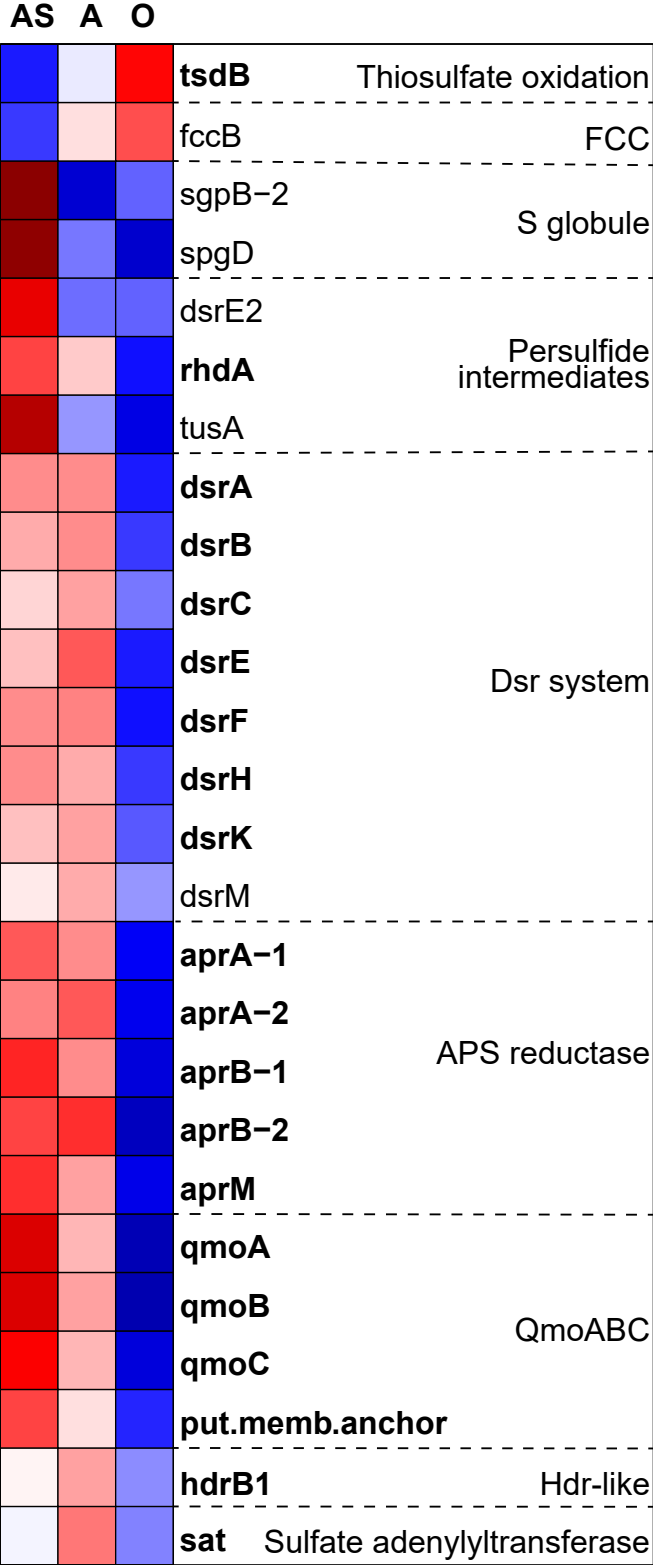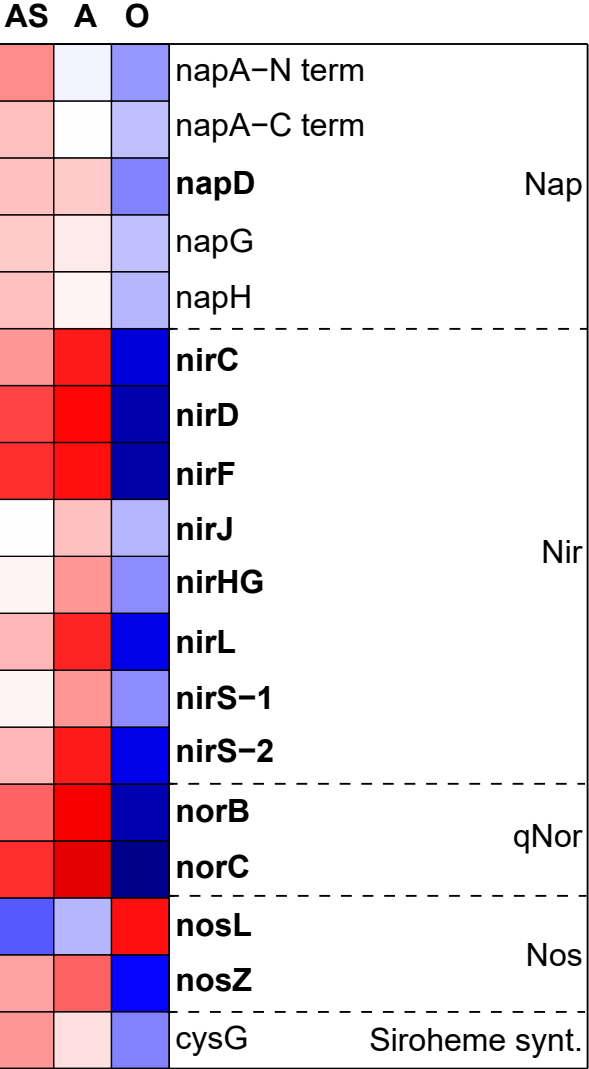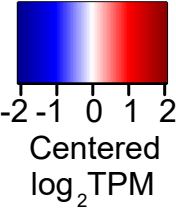**B**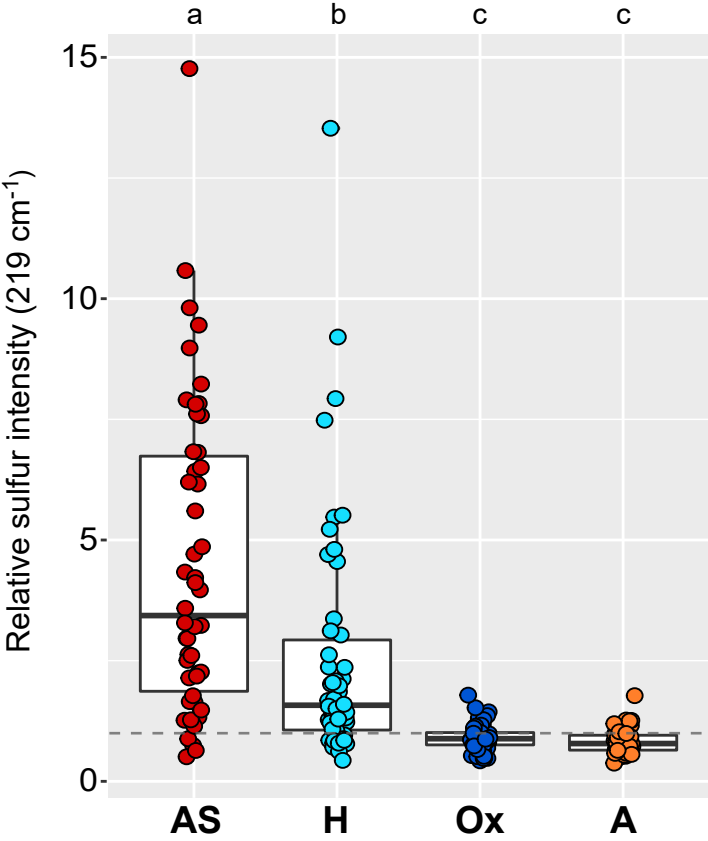

Supplement: FIG S1 [file msystems.01186-20-sf001.pdf]

**A**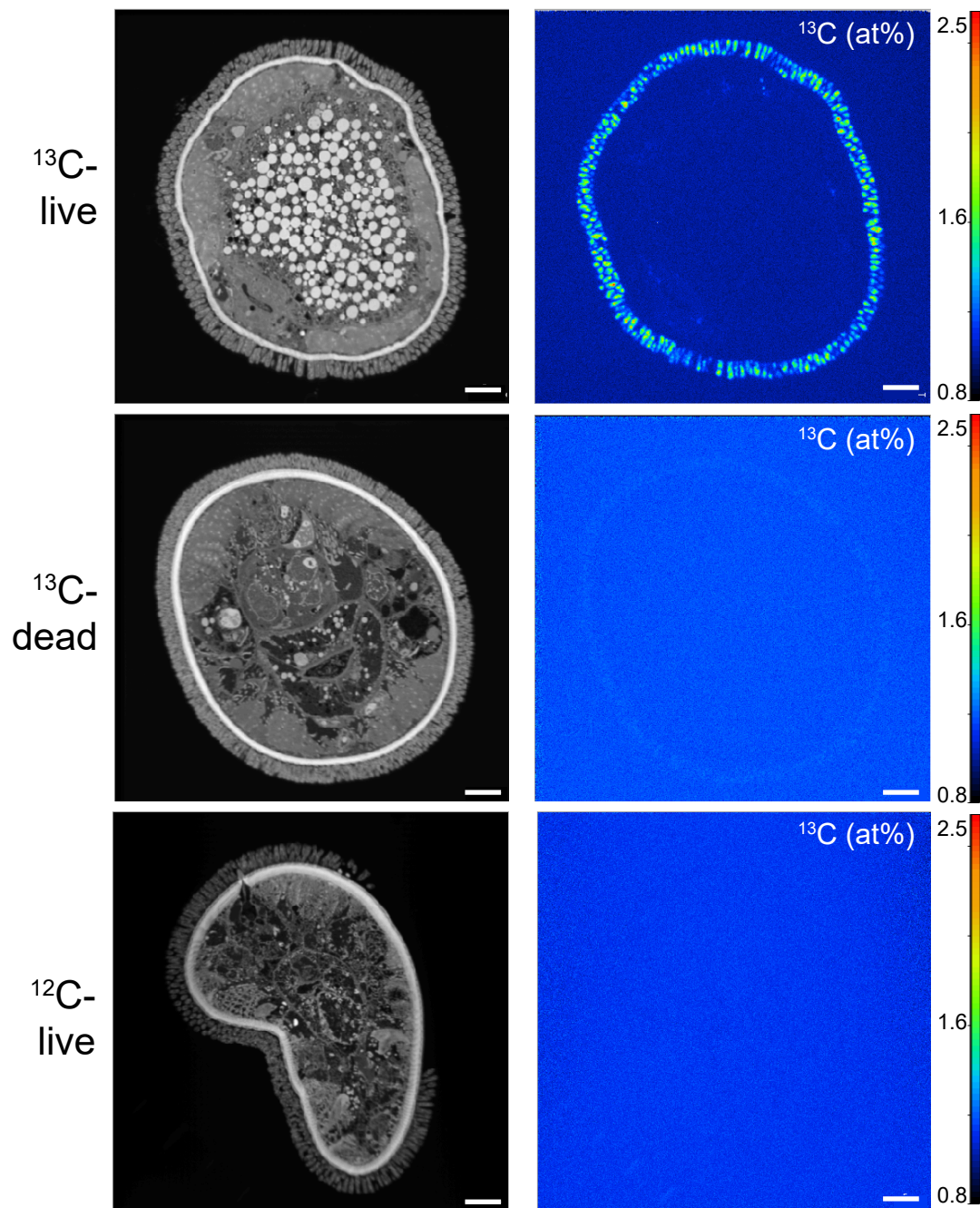**B**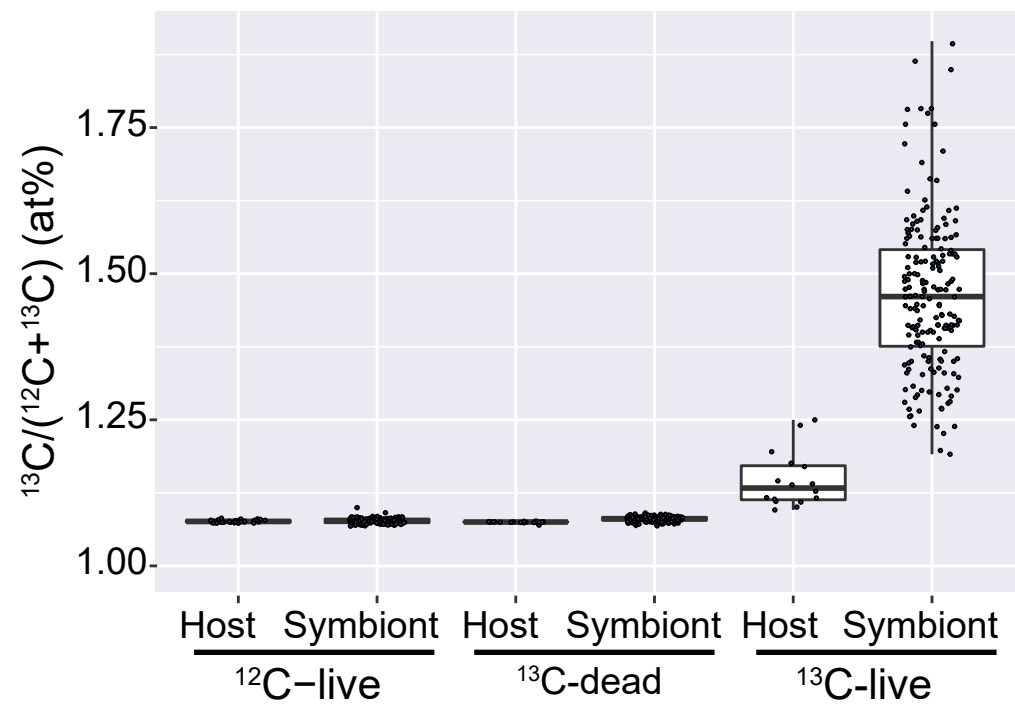

Supplement: FIG S3 [file msystems.01186-20-sf003.pdf]

**A**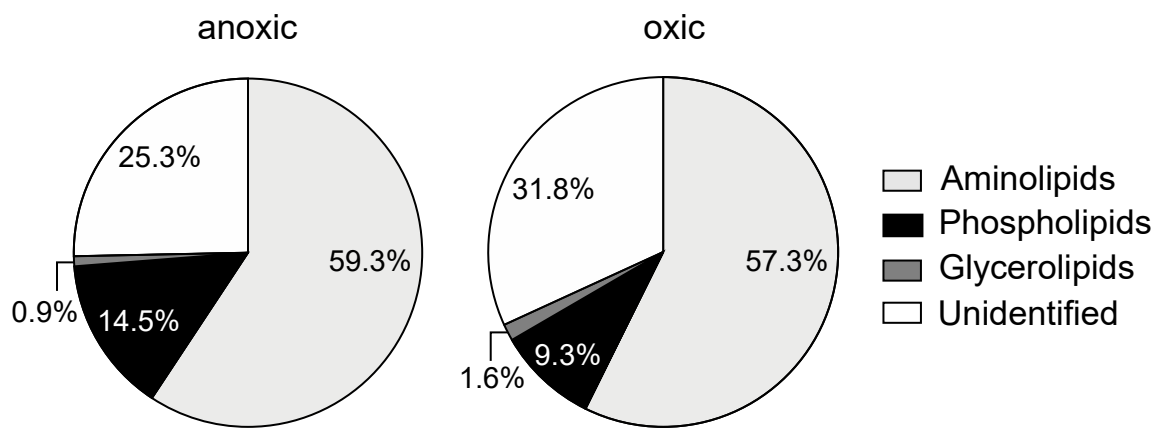**B**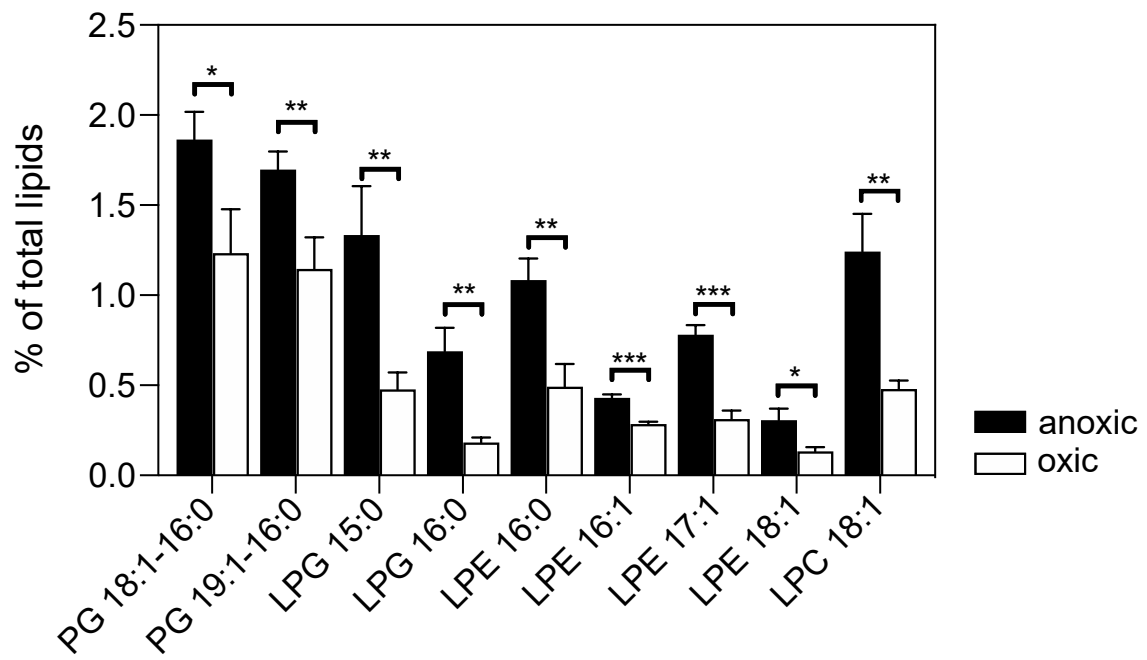

Supplement: FIG S2 [file msystems.01186-20-sf002.pdf]

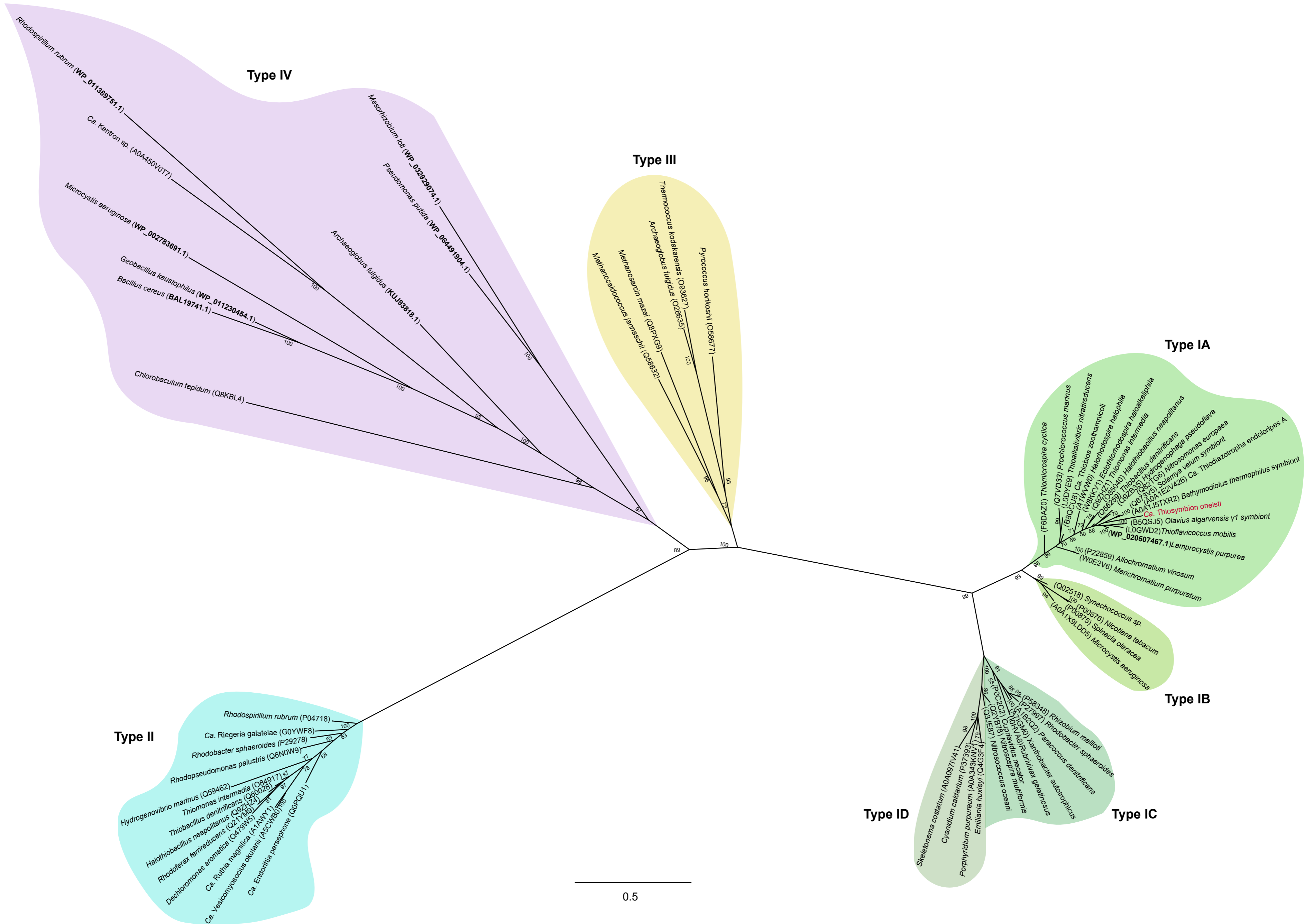

Supplement: FIG S4 [file msystems.01186-20-sf004.pdf]
