# Supplementary material for: Anaerobic Sulfur Oxidation Underlies Adaptation of a Chemosynthetic Symbiont to Oxic-Anoxic Interfaces
Source: mSystems. 2021 May 26;6(3):e01186-20. doi: 10.1128/mSystems.01186-20 (PMC8269255; doi:10.1128/mSystems.01186-20)
Supplement: FIG S5 [file msystems.01186-20-sf005.pdf]

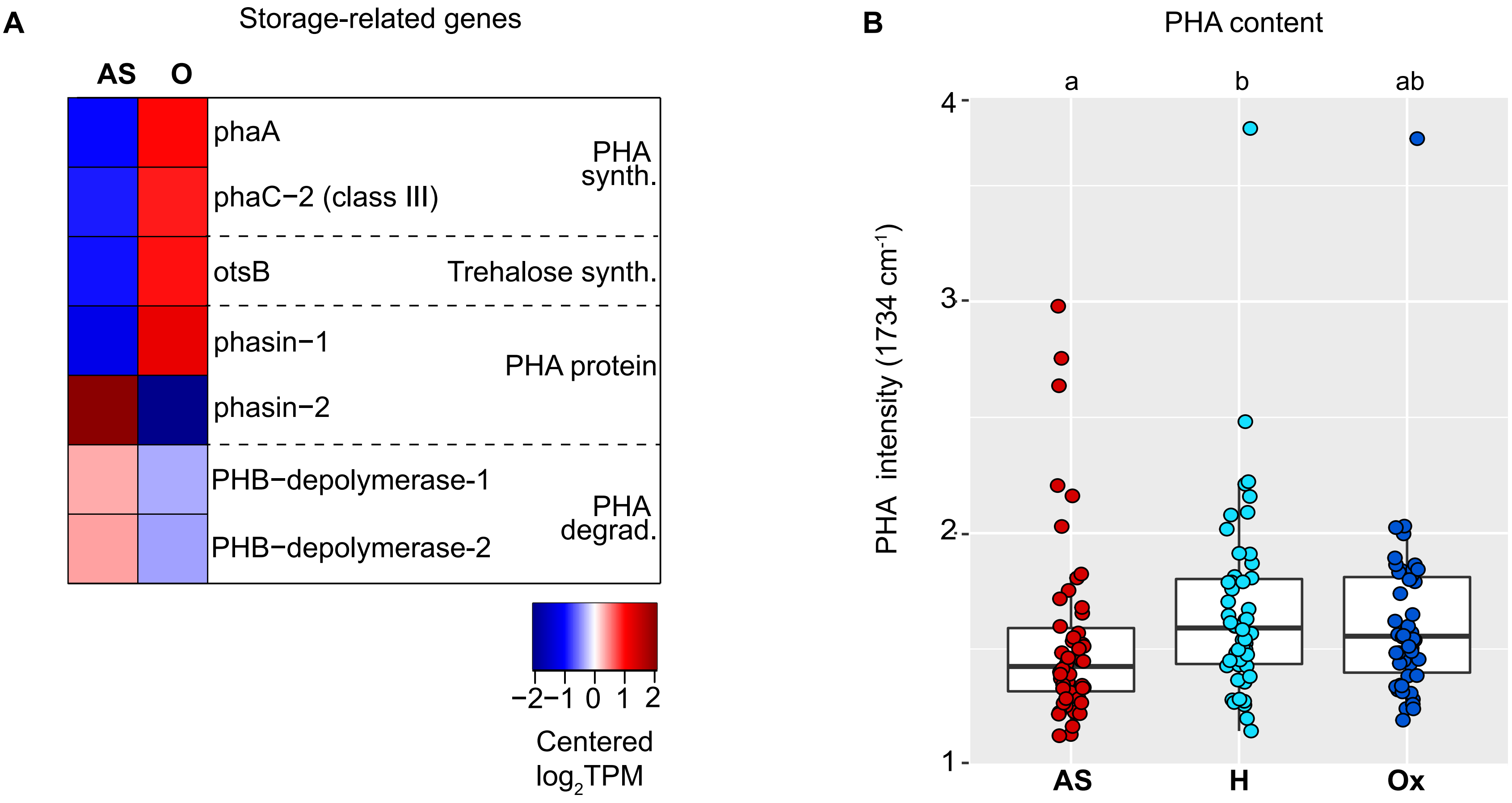
A
Storage-related genes
AS
O
phaA
phaC−2 (class III)
PHA synth.
otsB
Trehalose synth.
phasin−1
phasin−2
PHA protein
PHB−depolymerase-1
PHB−depolymerase-2
PHA degrad.
−2 −1 0 1 2
Centered log₂TPM
B
PHA content
a
b
ab
PHA intensity (1734 cm⁻¹)
1
2
3
4
AS
H
Ox
